# OsFH15, a class I formin, interacts with microfilaments and microtubules to regulate grain size via affecting cell expansion in rice

**DOI:** 10.1038/s41598-017-06431-5

**Published:** 2017-07-26

**Authors:** Tiantian Sun, Shanwei Li, Haiyun Ren

**Affiliations:** Key Laboratory of Cell Proliferation and Regulation Biology of Ministry of Education, College of Life Science, Beijing Normal University, Beijing, 100875 China

## Abstract

Grain size is an important agronomic trait determining rice yield and is mainly restricted by spikelet hull size. However, it remains largely unknown how the spikelet hull size is regulated. In this study, OsFH15, a class I formin protein in *Oryza sativa*, was found to be able to regulate the size of cells and spikelet hull. *OsFH15*-Cas9 and *OsFH15*-RNAi mutants had decreased grain size with reduced cell length, cell width and cell area of inner epidermal cells of the lemma compared with wild-type plants. By contrast, *OsFH15*-overexpressed plants had increased grain size with larger cells, as well as more abundant microtubules (MTs) and actin filaments (AFs) arrays. *OsFH15* was mainly expressed in shoot apical meristem (SAM), spikelets, spikelet hulls and seeds in rice. *In vitro* biochemical experiments showed that OsFH15 can efficiently nucleate actin polymerization with or without profilin, can cap the barbed end of AFs, and can bind and bundle both AFs and MTs. OsFH15 can also crosslink AFs with MTs, and preferentially bind MTs to AFs. These results demonstrated that OsFH15 played an important role in grain-size control by affecting cell expansion through regulating AFs and MTs.

## Introduction

Rice (*Oryza sativa*) is one of the most important staple food crops for nearly half of the world population. Improving grain yield is essential in rice-breeding programs^1^. Grain yield can be determined by grain size, which is in turn determined by grain width, grain length and grain thickness^2^. Many genes controlling rice-grain size have been successfully cloned in rice^3^. Numerous studies show that rice-grain size is controlled by multiple pathways but only a few size-associated genes have been functionally characterized to date.

In angiosperms, double-fertilization results in the formation of embryo and endosperm^4^. The maternal integument develops into seed coat enveloping the embryo and endosperm^5^. Embryo, endosperm and seed coat communicate with each other to ensure coordinated growth and development, thereby determining the final seed size. In *Arabidopsis*, mutations of EFRONIA (*EFR*), a plasma membrane receptor kinase, result in few but large seeds due to increased cell elongation in seed coats^6^. Overexpression of *EOD3*, encoding *Arabidopsis* cytochrome P450/CYP78A6, dramatically increases seed size by promoting both cell proliferation and cell expansion in embryo and maternal integuments^7^. In addition to endosperm growth or grain filling playing crucial roles in rice-grain size, the spikelet hull (consisting of a palea and a lemma) also set an upper limit to the final grain size^8, 9^. Grain size is further regulated by genes that affect cell number and/or cell size in palea and lemma. *GW2* and *GW5*/*qSW5* participate in the ubiquitin-proteasome pathway to modulate the cell number of spikelet hull^8–10^. *GW8*/*OsSPL16* affects cell proliferation to regulate spikelet hull size^11^. These studies indicate that the cell number of rice spikelet hull is controlled by various molecular pathways. POSITIVE REGULATOR OF GRAIN LENGTH 1 (PGL1) is a positive regulator inhibiting ANTAGONIST OF PGL1 (APG). The antagonistic pair of PGL1 and APG is involved in determining grain length by controlling cell length in spikelet hull^12^. Overexpression of BIG GRAIN1 (*BG1*), which is involved in auxin response and transport, leads to significantly increased grain size due to increased cell division and cell expansion in spikelet hulls^13^. Despite these genes having been identified to influence spikelet hull cell size, the biochemical and molecular mechanisms of regulating spikelet hull cell-size remain largely unknown.

The cytoskeleton, which includes the microtubule (MT) and actin filament (AF) systems, plays important roles in plant development and morphogenesis by cell-expansion regulation. For example, loss of function of OsKinesin-13A, an active MT depolymerase, reduces the transverse orientation and turnover of MTs and inhibits cell elongation, resulting in spikelet hull-length reduction^14^. The simultaneous downregulation of ACTIN2 and ACTIN7 inhibits cell expansion and elongation of *Arabidopsis* leaves and hypocotyls^15^. However, the molecular mechanisms by which AFs and MTs regulate these physiological processes require further investigation.

Several members of actin nucleating protein formins reportedly regulate both AFs and MTs in plants^16^. Similar to formins in fungi and animals, plant formins contain two highly conserved domains, the Pro-rich domain FH1 and the formin homology domain FH2^17^. The former binds profilin or actin/ profilin complexes to promote actin polymerization from the barbed end^18^. The latter nucleates new AFs as a dimer, binds to AFs, and regulates the organization of AF and MT^19–21^. In *Arabidopsis*, the class I formin AtFH1 affects actin organization directly and modulates MT dynamics^22, 23^. Moreover, the class I formin AtFH4 interacts with AFs directly and binds MTs through a plant-specific GOE domain^20^. The class II formin AtFH14 can also bind and bundle AFs and MTs *in vitro*, and is involved in plant-cell division^21^. Biochemical assays have further demonstrated that the class II formin AtFH16 can bind and bundle AFs and MTs^24^. In rice, the class II formin OsFH5 can also interact with both AFs and MTs *in vitro*, as well as participate in plant morphology^25^. In addition, another class I formin OsFH1 can regulate root-hair elongation^26^. However, little is known about whether OsFH1 interacts with AFs and/or MTs. To date, only 2 out of 16 formins have been investigated, thereby implying the significance of exploring the physiological functions of other formins in rice.

Here, we identified and characterized a previously undiscovered rice class I formin OsFH15 (LOC_Os09g34180). We found that *Osfh15* mutants and *OsFH15*-overexpressed plants had the decreased and increased grain size with less and more abundant cytoskeleton, respectively. *In vitro* biochemical experiments demonstrated that OsFH15 was the first formin to bind MTs to AFs simultaneously in rice and was the first plant class I formin to crosslink AFs with MTs. These findings showed that OsFH15 was a new positive regulator of grain size by controlling the AF and MT cytoskeleton systems.

## Results

### Generation of OsFH15 Mutations and Phenotype of the Mutants

**Figure 1:**
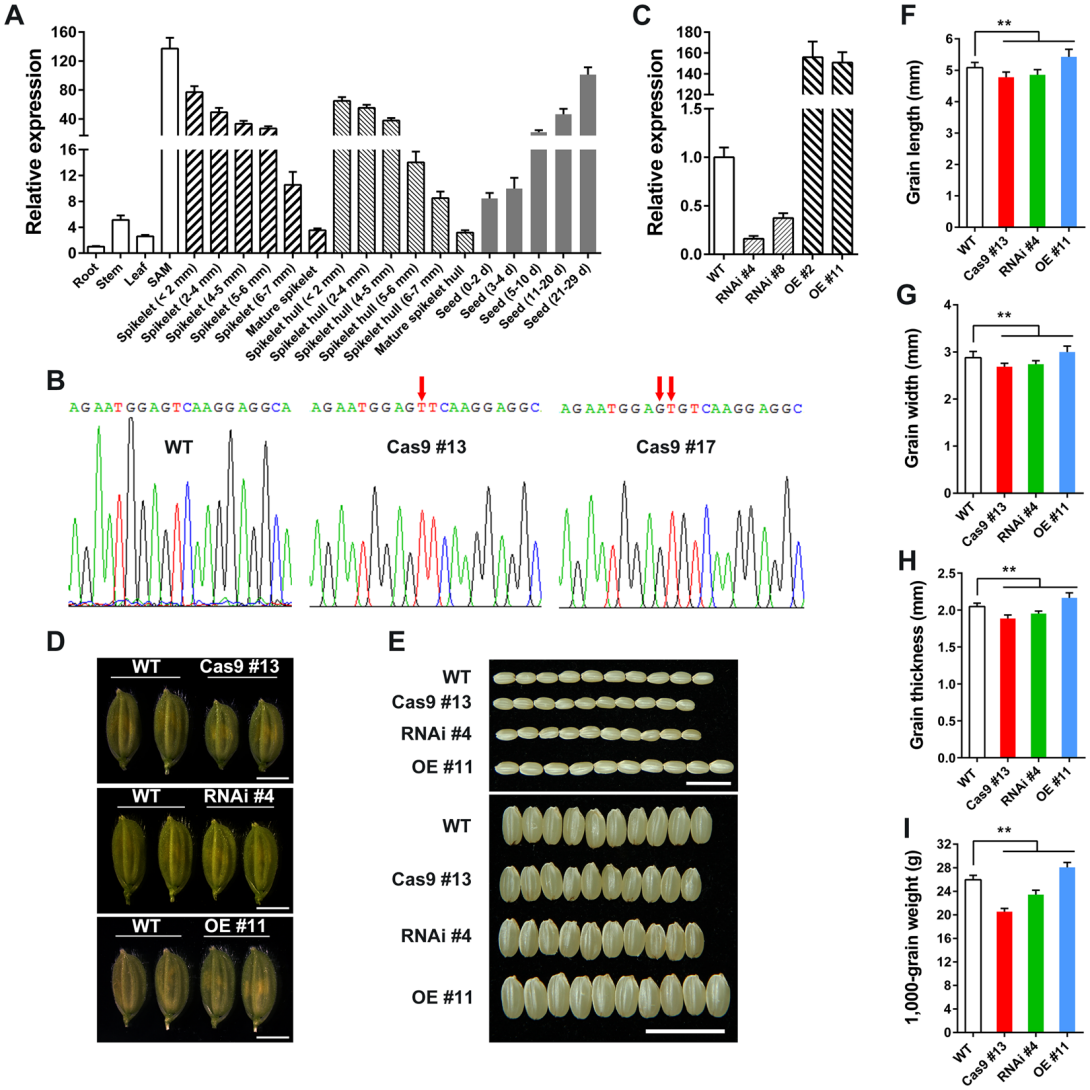
Phenotype with Overexpression and Repression of *OsFH15*. (**A**) qRT-PCR analysis of *OsFH15* expression pattern in various tissues. (**B**) SANGER sequencing chromatography showing the mutations in *Osfh15* mutant. (﻿**C**﻿) qRT-PCR analysis of *OsFH15* expression from 14 d-old seedling in wild-type and transgenic plants, *UBQ5* as control. (**D**) Spikelets of WT, Cas9 #13, RNAi #4 and OE #11 before heading. Bar = 5 mm. (**E**) Grains of WT, Cas9 #13, RNAi #4 and OE #11. Bar = 1 cm. (**F**) to (**I**) Grain length (**F**), Grain width (**G**) and Grain thickness (**H**) were determined by vernier depth. 1,000-grain weight (**I**) was weighed. Data are represented as mean ± SEM (n ≥ 100 in F-H, n = 15 in I). *P < 0.05, **P < 0.01, Student’s *t* tests were used to generate *P* values.

The expression patterns of *OsFH15* were analyzed by qRT-PCR analysis. Results revealed that *OsFH15* was mainly expressed in shoot apical meristem (SAM), young spikelet, young spikelet hull and seeds, *OsFH15* expression decreased with spikelet development and increased with seed development after pollination (Fig. 1A).

To investigate the biological function of OsFH15, we explored the CRISPR/Cas9 system to generate *OsFH15* mutants. A 23 bp nucleotide sequence targeting the coding-sequence regions of *OsFH15* was selected and ligated in one binary vector, which was then transformed into the wild-type rice Hwayoung by *Agrobacterium*-mediated transformation to edit *OsFH15* gene. In the T1 generation, we obtained 30 positive transgenic plants through hygromycin resistance. In the T2 generation, we analyzed target sites with the deletion of *OsFH15* gene by using PCR and Sanger sequencing. We identified two independent mutant lines, namely, Cas9 #13 and Cas9 #17 (Fig. 1B). The Cas9 editing target was located at 1185 bp and 1207 bp in the coding region of *OsFH15*. The 1 bp and 2 bp insertion in the Cas9 editing targets were found in Cas9 #13 and Cas9 #17, respectively (Fig. 1B). The insertions led to reading frame shifts, thereby producing stop codons at 1357 bp with 452 amino acids (a.a.) and 1285 bp with 428 a.a. in Cas9 #13 and Cas9 #17, respectively. To exclude the influence of *Cas9* gene, we isolated Cas9 #13 and Cas9 #17 by screening for hygromycin resistance, and only nonhygromycin resistant mutants were retained and used for further investigation. To further uncover the potential growth and development functions of OsFH15, we generated *OsFH15* overexpression (OE) and RNA-interference (RNAi) transgenic plants. The relative expression levels of *OsFH15* were examined in 14 d-old seedlings from wild-type and transgenic plants, with *UBIQUITIN5* (*UBQ5*) serving as the reference gene. Results revealed that *OsFH15* expression was significantly increased in OE plants and decreased in RNAi plants (Fig. 1C).

**Figure 2:**
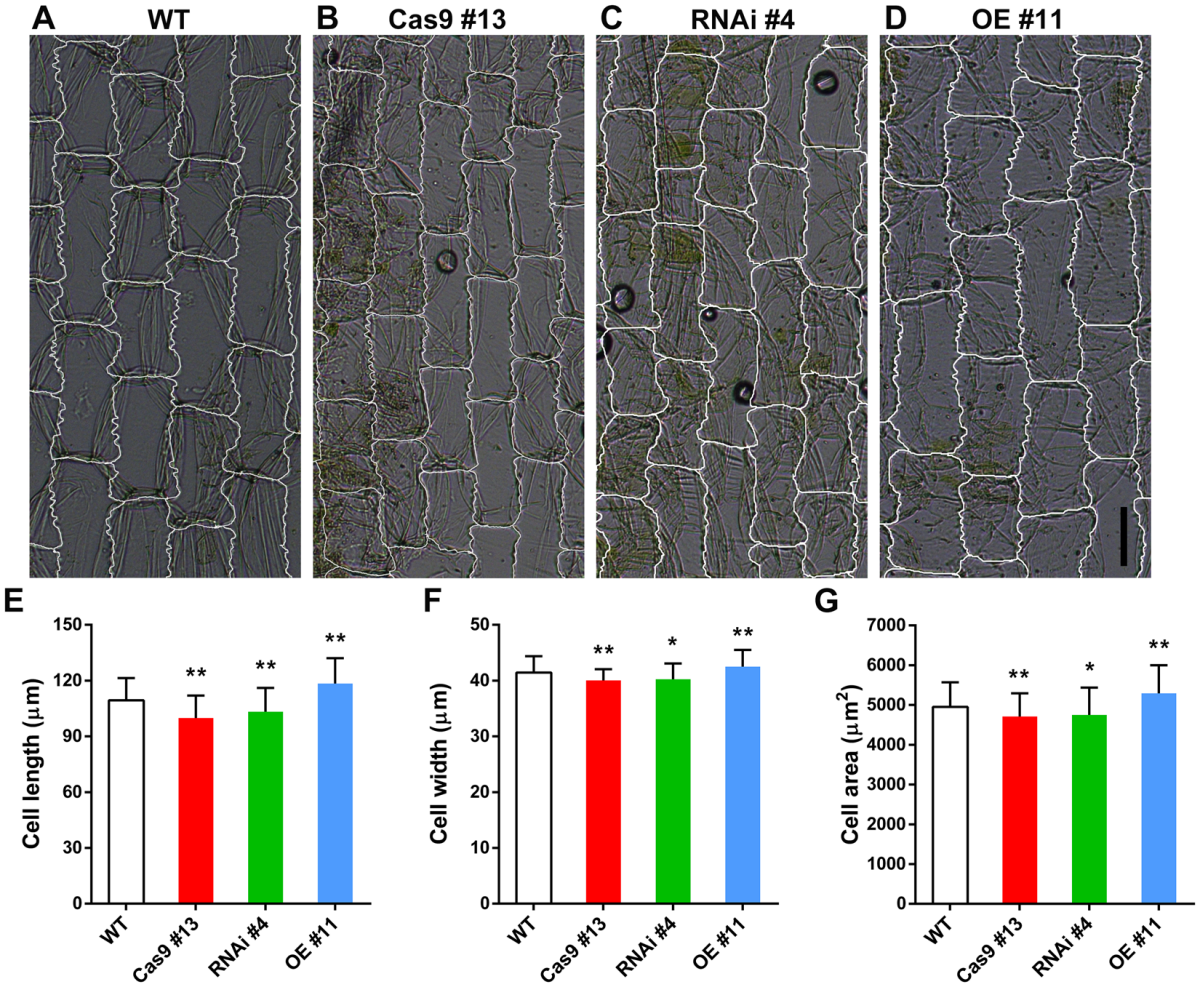
The Effect of OsFH15 on Cell Size in Lemma. (**A**) to (**D**) Images of inner epidermal cells of the lemma. Bar in (**D**) = 50 μm. (**E**) to (**G**) Statistical data of the cell length (**E**), cell width (**F**) and cell area (**G**) of the lemma inner epidermal cells, respectively. Data are represented as mean ± SEM (n ≥ 200 in **E–G**). *P < 0.05, **P < 0.01, Student’s *t* tests were used to generate *P* values.

Compared with wild-type rice, we observed decreased grain length, grain width, grain thickness and 1,000-grain weight by 3.28%, 6.64%, 7.91% and 20.95%, respectively, for Cas9 #13 line; the corresponding values were 2.48%, 4.80%, 6.16% and 9.78%, respectively, for RNAi #4 line (Fig. 1E–I). For *OsFH15*-overexpressing plants (OE #11), grain length (2.87%), grain width (4.21%), grain thickness (2.93%) and 1,000-grain weight (8.09%) significantly increased compared with the wild type (Fig. 1E–I). Cas9 #17, RNAi #8 and OE #2 exhibited seed phenotypes similar to those of Cas9 #13, RNAi #4 and OE #11, respectively (Supplemental Fig. 1). These results suggested that OsFH15 can regulate grain size. In rice, grain size is rigidly controlled by spikelet hull volume (size) and grain filling^8, 27^ (Supplemental Fig. 2A,B). The spikelet hulls of Cas9 #13 and RNAi #4 were smaller than those of the wild type. However, OE #11 plants exhibited larger spikelet hulls (Fig. 1D). Furthermore, spikelet hull volume was determined by the cell number and/or cell size of spikelet hull. To characterize seed phenotype in detail, we observed the inner epidermal cells of mature lemmas. Results revealed that the length, width and area of inner epidermal cells of lemmas decreased by 8.74%, 3.43% and 4.85% in Cas9 #13, decreased by 5.58%, 2.87% and 4.04% in RNAi #4; and increased by 8.23%, 2.51% and 6.91% in OE #11 compared with those in the wide type (Fig. 2). From these observations, we concluded that OsFH15 positively regulated grain size by controlling cell size, which led to longitudinal and latitudinal changes in grain.

Compared with wild-type plants, Cas9 #13 and RNAi #4 anthers appeared shorter (Supplemental Fig. 3A,B,D,E,K) with smaller cells (Supplemental Fig. 3G–I,L). By contrast, OE #11 anthers were longer (Supplemental Fig. 3C,F,K) and had larger cells (Supplemental Fig. 3G,J,L). These results indicated that OsFH15 was a positive regulator for cell size. Additionally, transgenic plants displayed a moderate dwarf phenotype of plant height (Supplemental Fig. 4A). The average effective tiller number and grain yield per plant of transgenic plants also decreased compared with those of wild-type plants (Supplemental Fig. 4C,D). However, the number of grains per tiller had no obvious change (Supplemental Fig. 4B). These data indicated that inhibition and overexpression of *OsFH15* induced negative effect on yield production.

### AF and MT Organization Are Impaired in OsFH15 Mutants

**Figure 3:**
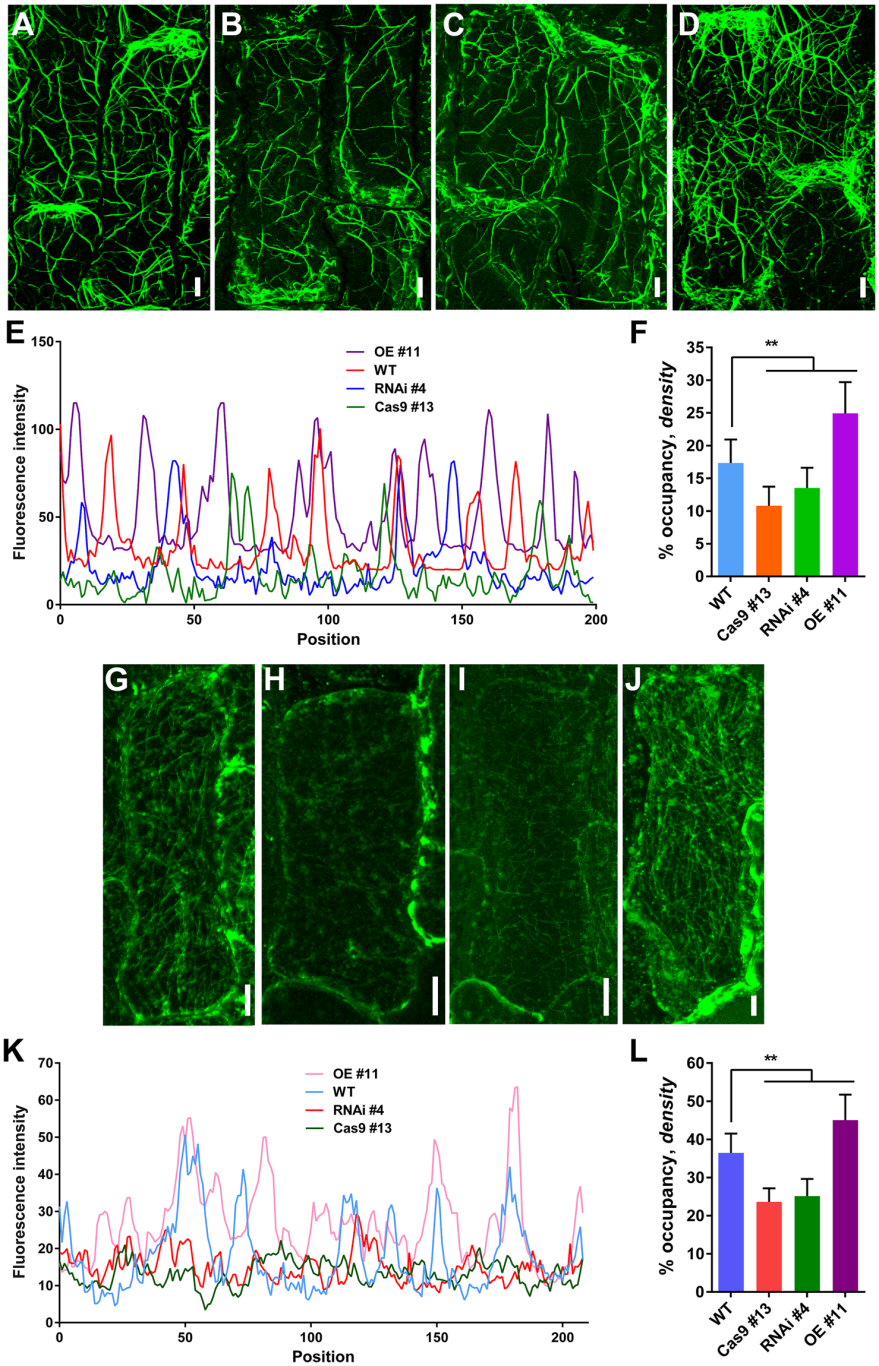
AF and MT Organization in Wild-Type and Transgenic Cells. (**A**) to (**D**) AF organization was stained by Alexa Fluor 488-phalloidin, (**A**) WT, (**B**) Cas9 #13, (**C**) RNAi #4, (**D**) OE #11. (**G**) to (**J**) MTs were stained with tubulin antibody, (**G**) WT, (**H**) Cas9 #13, (**I**) RNAi #4, (**J**) OE #11. (**A**) to (**D**) and (**G**) to (**J**) The cells from the inner epidermal cells of lemmas. (**E**) and (**K**) Fluorescence intensities corresponding to the regions of cell middle part in (**A**) to (**D**) and (**G**) to (**J**), respectively. This cell middle part was marked in Supplemental Fig. 2C with white line. (**F**) and (**L**) Average AF and MT density were measured and binned for the same regions in (**A**) to (**D**) and (**G**) to (**J**), respectively. Bar in (**A**) to (**D**) and (**G**) to (**J**) = 10 μm. Data are represented as mean ± SEM (n ≥ 50 in F and L). *P < 0.05, **P < 0.01, Student’s *t* tests were used to generate *P* values.

Cytoskeleton is related to cell size and formins can regulate actin cytoskeleton assembly and organization^28^. To assess AF arrangement in the epidermal cells of rice lemmas, we fixed the inner epidermal cells of lemmas, which were stained with Alexa Fluor 488-phalloidin, a dye that specifically labels actin cytoskeleton. In the wild type, the inner epidermal cells of lemmas displayed a fine AF network with abundant filaments (Fig. 3A). By contrast, the fluorescence of phalloidin staining was weaker in Cas9 #13 and RNAi #4 but much stronger in OE #11 than that in the wild type under identical staining and image-acquisition conditions (Fig. 3B–D). We also measured the percentage occupancy (density) and AF bundling to assess actin-array organization following *OsFH15* mutation. Density decreased in Cas9 #13 and RNAi #4 but increased in OE #11, revealing that the amount of AF was decreased in Cas9 #13 and RNAi #4 but increased in OE #11 (Fig. 3F). Fluorescence intensity exhibited peaks in wild-type cells, which corresponded with the thick actin cables. Such peaks were compromised in Cas9 #13 and RNAi #4 cells but enhanced in OE #11 cells (Fig. 3E). In addition, changes in the density and fluorescence intensity of AFs in anther cells were similar to those in lemma cells of wild-type and transgenic plants (Supplemental Fig. 5A–F). In summary, OsFH15 played an important role in AF organization in rice.

The MT system is highly dynamic and plays a vital role in many important cellular processes in plant cells. The components of the MT system are reportedly critical to plant architecture^25, 29, 30^ and cell-shape maintenance^31^. To observe MT arrangements in mutants, we stained MTs in lemma cells with anti-β-tubulin antibody. The lemma cells of Cas9 #13 and RNAi #4 contained fewer MT arrays contrasted with the well-developed and complex MT network in the wild type (Fig. 3G–I). However, the lemma cells of OE #11 had more abundant MT arrays compared with the wild type (Fig. 3J). In contrast to the wild type, fluorescence intensity and density were lower in Cas9 #13 and RNAi #4 cells but higher in OE #11 cells (Fig. 3K,L). Moreover, the alterations in density and fluorescence intensity of MTs in anther cells were similar to those in lemma cells of wild-type and transgenic plants (Supplemental Fig. 5G–L). These results indicated that OsFH15 also regulated MT organizations in rice.

Furthermore, it was shown that the amounts of total actin and tubulin remained almost constant in WT and transgenic plants (Supplemental Fig. 6), implying that changes in AFs and MTs level were not due to changes in amounts of total actin and tubulin but due to the function of OsFH15.

### OsFH15 Nucleates Actin Assembly

OsFH15 was identified based on the sequence analysis of the rice genome. The gene contained four exons and three introns, encoding a 2367 bp mRNA translating 788 a.a. with an estimated molecular mass of 84.5 kDa. OsFH15 was a class I formin^32, 33^ containing a signal peptide (SP, a.a. 1–18), Pro-rich domain (a.a. 44–86), and transmembrane domain (TM, a.a. 148–168) at its N terminus; a typical FH1 domain (a.a. 257–297), a characteristic FH2 domain (a.a. 340–768) at its C-terminal halves (Supplemental Fig. 7A).

**Figure 4:**
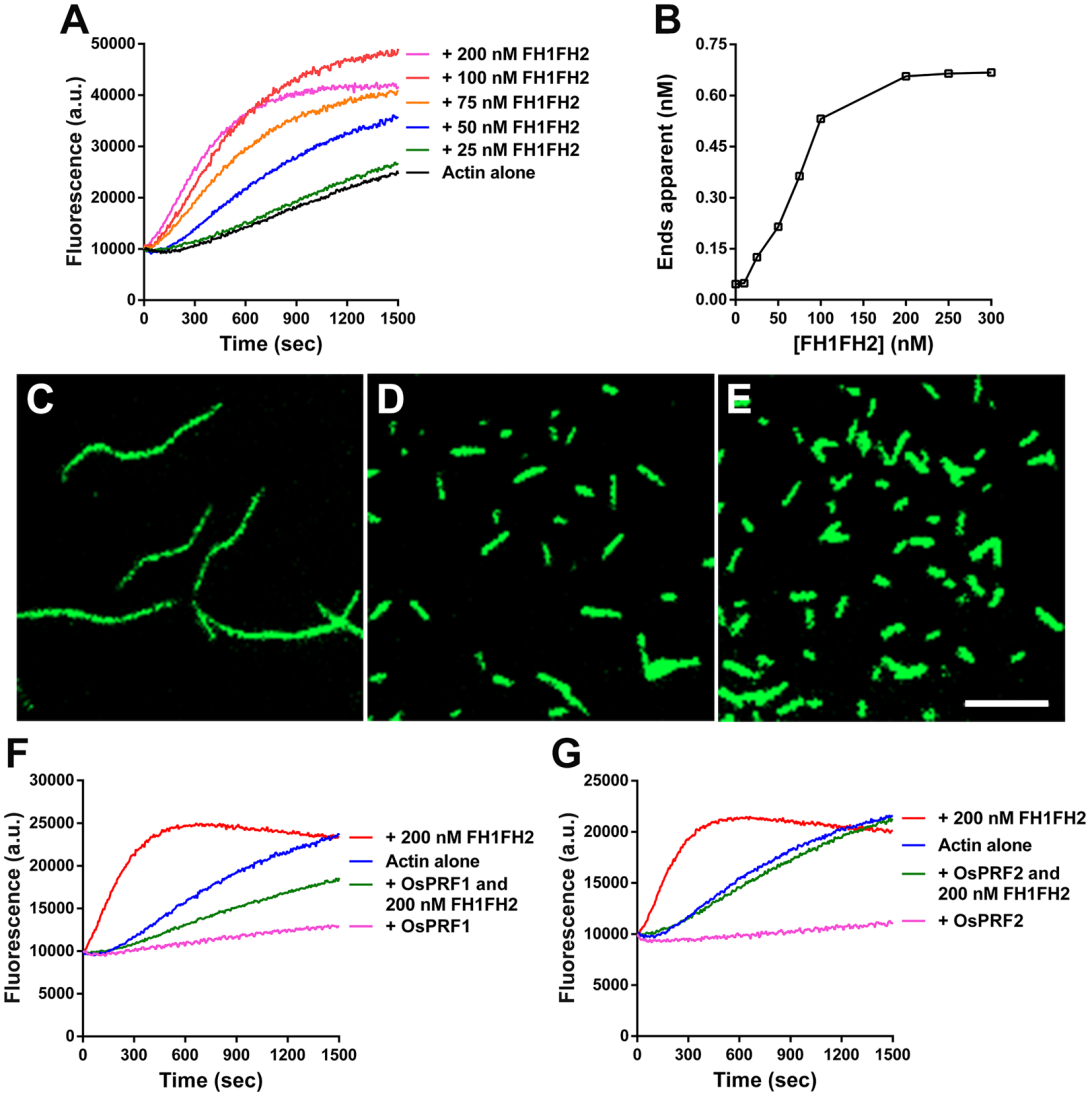
FH1FH2 Nucleates Actin Polymerization. (**A**) Time course of actin polymerization in the presence of FH1FH2 monitored by pyrene fluorescence. Different concentrations of FH1FH2 were added to 2 μM actin (10% pyrene labeled) before initiation of polymerization. a.u., arbitrary units. (**B**) Nucleation efficiency of FH1FH2. (**C**) to (**E**) Fluorescence image of Oregon-green-actin polymerization in the absence and presence of FH1FH2. (**C**) Oregon-green-actin alone, (**D**) Oregon-green-actin plus 100 nM FH1FH2, (**E**) Oregon-green-actin plus 200 nM FH1FH2. Bar in (**E**) = 5 μm. (**F**) Time course of actin polymerization in the presence of FH1FH2 and/or OsPRF1. (**G**) Time course of actin polymerization in the presence of FH1FH2 and/or OsPRF2.

To examine the chemical properties of OsFH15 in actin and MT dynamics *in vitro*, we generated two truncated recombinant proteins containing the FH1FH2 domain (referred to as FH1FH2) and FH2 domain (referred to as FH2) of OsFH15. The two recombinant fusion proteins were expressed and purified from bacterial cells (Supplemental Fig. 7A,B). To determine whether OsFH15 nucleated actin assembly, we then examined the effects of FH1FH2 and FH2 on actin polymerization by using the pyrene-actin assay. Pyrene-labeled actin monomers (10% pyrene labeled) were incubated with various concentrations of FH1FH2 and FH2, and actin polymerization was monitored by pyrene fluorescence. Figure 4A shows that in the kinetic pyrene-actin assays, FH1FH2 decreased the initial lag phase of actin polymerization in a concentration-dependent manner, corresponding to its active nucleation activity. However, the active nucleation activity of FH2 was very weak (Supplemental Fig. 8A). The concentration of new barbed ends strongly depended on the FH1FH2 amount (Fig. 4B). To further investigate the ability of OsFH15 on actin assembly, we used total internal reflection fluorescence microscopy (TIRFM) to directly visualize actin polymerization from Oregon-green-actin. Obviously, the presence of FH1FH2 significantly increased the AF amount (Fig. 4C–E), thereby confirming its nucleation activity.

The majority of actin monomers are reportedly buffered by highly abundant profilin, and the actin/profilin complex exercises its physiology functions in plants^34^. Accordingly, we determined whether OsFH15 can use the actin/profilin complex for efficient actin nucleation. Two profilins exist in rice, i.e., profilin 1 (OsPRF1) and OsPRF2. We purified OsPRF1 and OsPRF2 with poly-L-proline Sepharose (Supplemental Fig. 7C) and then used the two profilins in the nucleation experiment. As shown in Fig. 4F,G, in the presence of OsPRF1 or OsPRF2, FH1FH2 also showed active actin nucleating ability. However, FH2 still did not nucleate actin polymerization in the presence of rice profilins (Supplemental Fig. 8B,C). Our results indicated that OsFH15 can nucleate actin polymerization from actin or actin/profilin complex.

### OsFH15 Binds the Barbed End of AFs and Inhibits Elongation

**Figure 5:**
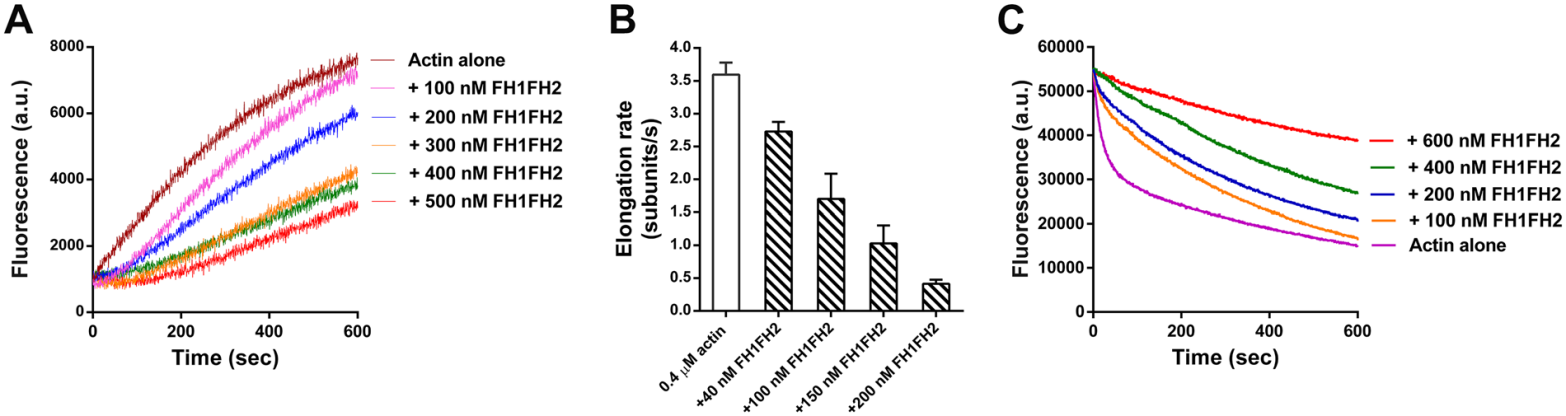
FH1FH2 Attaches to the AF Barbed End and Inhibits the Elongation. (**A**) Kinetics of elongation of the barbed-end of AFs in the presence of FH1FH2. Preformed AF seeds (0.8 μM) were incubated with various concentrations of FH1FH2 before addition of 1 μM pyrene-actin monomers. a.u., arbitrary units. (**B**) Average elongation rates of AFs with or without FH1FH2 on the barbed end at different concentrations. Data are represented as mean ± SEM, n ≥ 30, N ≥ 3. (﻿**C**﻿) Kinetics of AF depolymerization in the presence of FH1FH2. FH1FH2 was incubated with 5 μM AFs (60% pyrene-labeled) for 5 min before dilution of the solution 25-fold in G buffer.

As one of their general features, several plant formins feature barbed-end capping activity on AFs. To examine barbed-end dynamics in the presence of OsFH15, we performed the seeded AF elongation assay. As shown in Fig. 5A, initial elongation rate decreased with increasing concentration of FH1FH2. We also used TIRFM to directly examine barbed-end dynamics in the presence of OsFH15. The initial elongation rate of actin assembly was reduced by FH1FH2 in a concentration-dependent manner (Fig. 5B, Supplemental Movie 1). These results indicated that FH1FH2 capped barbed ends of AFs. Reduction of depolymerization rate reflected barbed-end capping activity because depolymerization rate of barbed ends is more than 20-fold faster than that of pointed ends^35^. We performed the dilution-mediated AF depolymerization assay to examine capping activity of OsFH15. Initial depolymerization rate decreased in the presence of FH1FH2 in a concentration-dependent manner (Fig. 5C). Our results implied that OsFH15 can stabilize AFs because of its barbed-end capping activity.

Several studies showed that profilin can increase rates of AF elongation associated with FH1FH2 domain of formins^18, 36, 37^. We selected OsPRF1 to investigate interaction effects of profilins and OsFH15 on regulating the elongation rate of AFs. Using TIRFM, we measured activities of OsFH15 in the presence of OsPRF1 *in vitro*. In the presence of FH1FH2, elongation rate of actin reached ~0.4 subunits/s (Supplemental Fig. 7F, Supplemental Movie 2). By contrast, when OsPRF1 was added to the system, elongation rate for actin/profilin increased to ~1.4 subunits/s in the presence of FH1FH2 (Supplemental Fig. 7F, Supplemental Movie 2). These results demonstrated that OsPRF1 can promote rates of FH1FH2-mediated filament elongation.

### OsFH15 Binds and Bundles AFs and MTs

**Figure 6:**
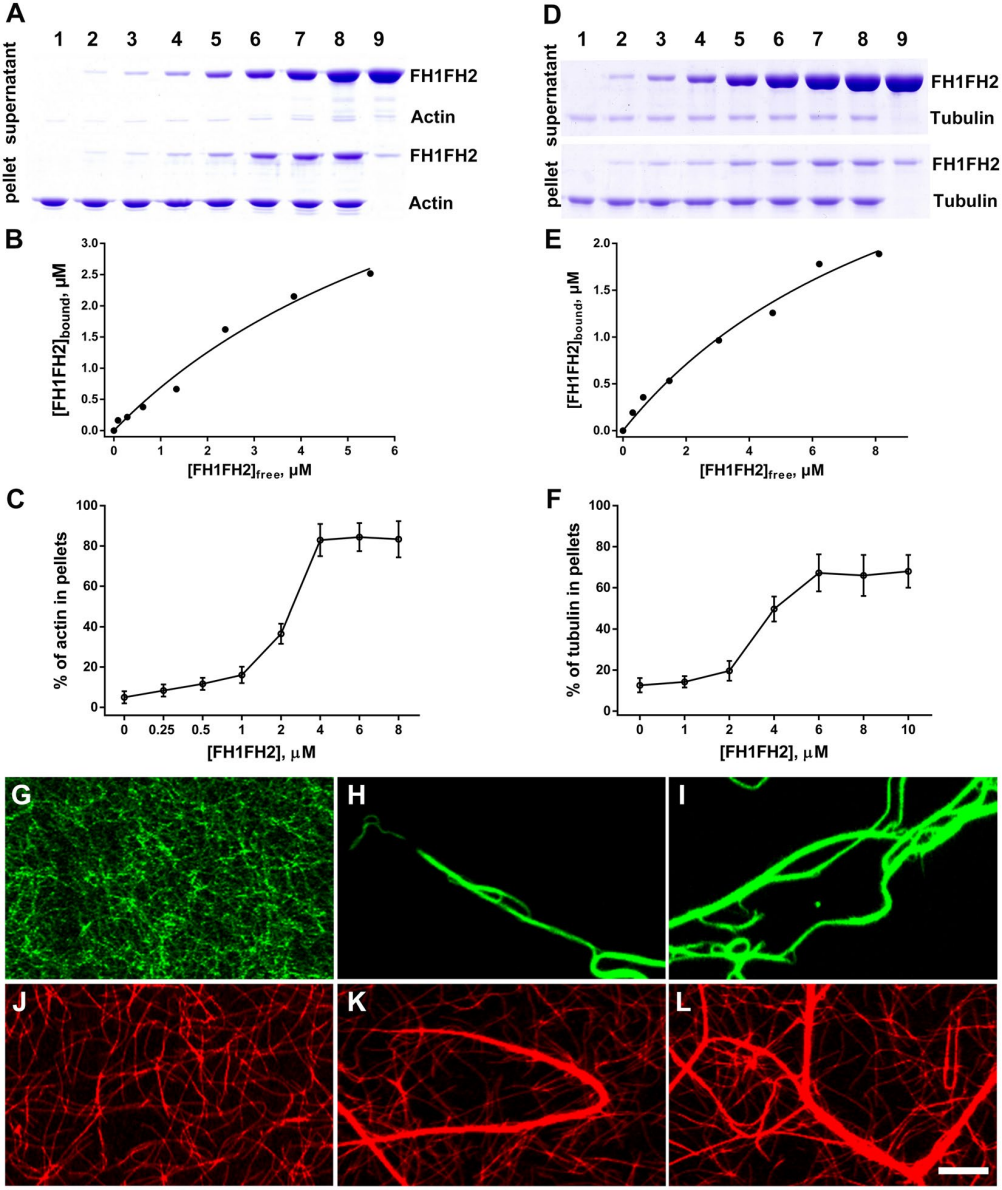
FH1FH2 Binds and Bundles AFs and MTs *in Vitro*. (**A**) A high-speed cosedimentation assay was performed to assess the binding between FH1FH2 and AFs. Lane 1, actin alone (3 μM); lanes 2–8, actin plus 0.25, 0.5, 1, 2, 4, 6, or 8 μM FH1FH2, respectively; Lane 9, FH1FH2 alone (8 μM). (**B**) Quantification of scanned SDS-PAGE gel from (**A**). The concentration of bound FH1FH2 was plotted against the concentration of free FH1FH2 and fitted with a hyperbolic function. For this representative experiment, the *Kd* was calculated to be 0.82 μM. (**C**) Percentage of AFs recovered in the low-speed pellet in different concentration of FH1FH2. (**D**) A high-speed cosedimentation assay was used to determine the binding affinity between FH1FH2 and MTs. Lane 1, tubulin alone (1 μM); lanes 2–8, tubulin plus 1, 2, 4, 6, 8, 10, or 12 μM FH1FH2, respectively; Lane 9, FH1FH2 alone (12 μM). (**E**) Quantification of scanned SDS-PAGE gel from (**D**). The concentration of bound FH1FH2 was plotted against the concentration of free FH1FH2 and fitted with a hyperbolic function. For this representative experiment, the *Kd* was calculated to be 1.02 μM. (**F**) Percentage of MTs recovered in the low-speed pellet in different concentration of FH1FH2. (**G**) to (**I**) Micrographs of AFs in the absence or presence of FH1FH2. (**G**) 0.5 μM AFs alone, (**H**) 0.5 μM AFs plus 500 nM FH1FH2, (**I**) 0.5 μM AFs plus 1 μM FH1FH2. (**J**) to (**L**) Micrographs of MTs in the absence or presence of FH1FH2. (**J**) 0.5 μM MTs alone, (**K**) 0.5 μM MTs plus 500 nM FH1FH2, (**L**) 0.5 μM MTs plus 1 μM FH1FH2. Bar in (**L**) = 10 μm.

We performed high-speed cosedimentation assay to test whether OsFH15 bound to AFs. As shown in Fig. 6A, FH1FH2 cosedimented with AFs in a FH1FH2 concentration-dependent manner, indicating that FH1FH2 can directly bind AFs. Based on three independent experiments, mean *Kd* value of FH1FH2 binding AFs measured 0.82 ± 0.06 µM (mean ± SD; Fig. 6B). FH2 exhibited weaker AF-binding ability than FH1FH2 with a mean *Kd* value of 1.51 ± 0.07 µM (Supplemental Fig. 9A,B). We next performed low-speed cosedimentation assay to test whether OsFH15 affected bundling of AFs. As negative controls, AFs and FH1FH2 alone were recovered mostly in supernatants. With addition of FH1FH2, amounts of actin in pellet increased in a concentration-dependent manner (Fig. 6C, Supplemental Fig. 10A), indicating bundling activity. FH2 also presented actin bundling ability (Supplemental Fig. 9C,D). When amounts of actin in pellet were saturated, lower concentration of FH1FH2 was noted in comparison with FH2 in fixed concentration of actin monomers, this result implied stronger bundling activity of FH1FH2.

Under fluorescence microscopy, we discovered that AFs failed to form actin bundles in absences of FH1FH2 or FH2 (Fig. 6G, Supplemental Fig. 9I). However, both FH1FH2 and FH2 organized individual AFs into long actin bundles (Fig. 6H,I, Supplemental Fig. 9I). TIRFM was used to confirm the truth of the actin bundling ability of FH1FH2, and observations showed that FH1FH2 initiated assembly and bundled AFs (Supplemental Movie 3). FH1FH2 still induced bundling of AFs in the presence of OsPRF1 (Supplemental Fig. 7D,E). Collectively, these results demonstrated that OsFH15 can efficiently bind to and bundle AFs via the FH2 domain *in vitro*.

Recently, several studies showed that formins not only bundle AFs^25, 38, 39^ but also regulate MT cytoskeleton^19–21, 25, 39^. We performed high-speed cosedimentation assay to test interactions between MTs and OsFH15. Small amounts of FH1FH2 were detected in pellet without MTs. However, the amount of sedimented FH1FH2 increased with addition of MTs (Fig. 6D). FH2 also yielded similar results (Supplemental Fig. 9E). These results confirmed that FH1FH2 and FH2 directly bound MTs *in vitro*. Mean *Kd* values reached 1.02 ± 0.05 µM for FH1FH2 (Fig. 6E) and 1.81 ± 0.09 µM for FH2 (Supplemental Fig. 9F), indicating that FH1FH2 bound MTs with higher affinity. We also performed low-speed cosedimentation assay to test whether OsFH15 bundled MTs. In the absence of FH1FH2 or MTs, most MTs or FH1FH2 appeared in supernatants, and percentage of MTs in pellet increased in a concentration-dependent manner with increasing amounts of FH1FH2 (Fig. 6F, Supplemental Fig. 10B), thus implying bundle formation. Compared with FH1FH2, FH2 can also bundle MTs but at a lower bundling efficiency (Supplemental Fig. 7G,H).

Effects of OsFH15 on MTs bundle formation were further confirmed by fluorescence microscopy. We observed that MTs were scattered individually without FH1FH2 (Fig. 6J), but MT bundles formed when FH1FH2 was added (Fig. 6K,L). Similar to FH1FH2, FH2 can also organize MTs into thick MT bundles (Supplemental Fig. 9I). We concluded that FH2 domain was vital for OsFH15 to bind and bundle MTs.

### OsFH15 Preferentially Binds MTs over AFs and Crosslinks AFs with MTs

**Figure 7:**
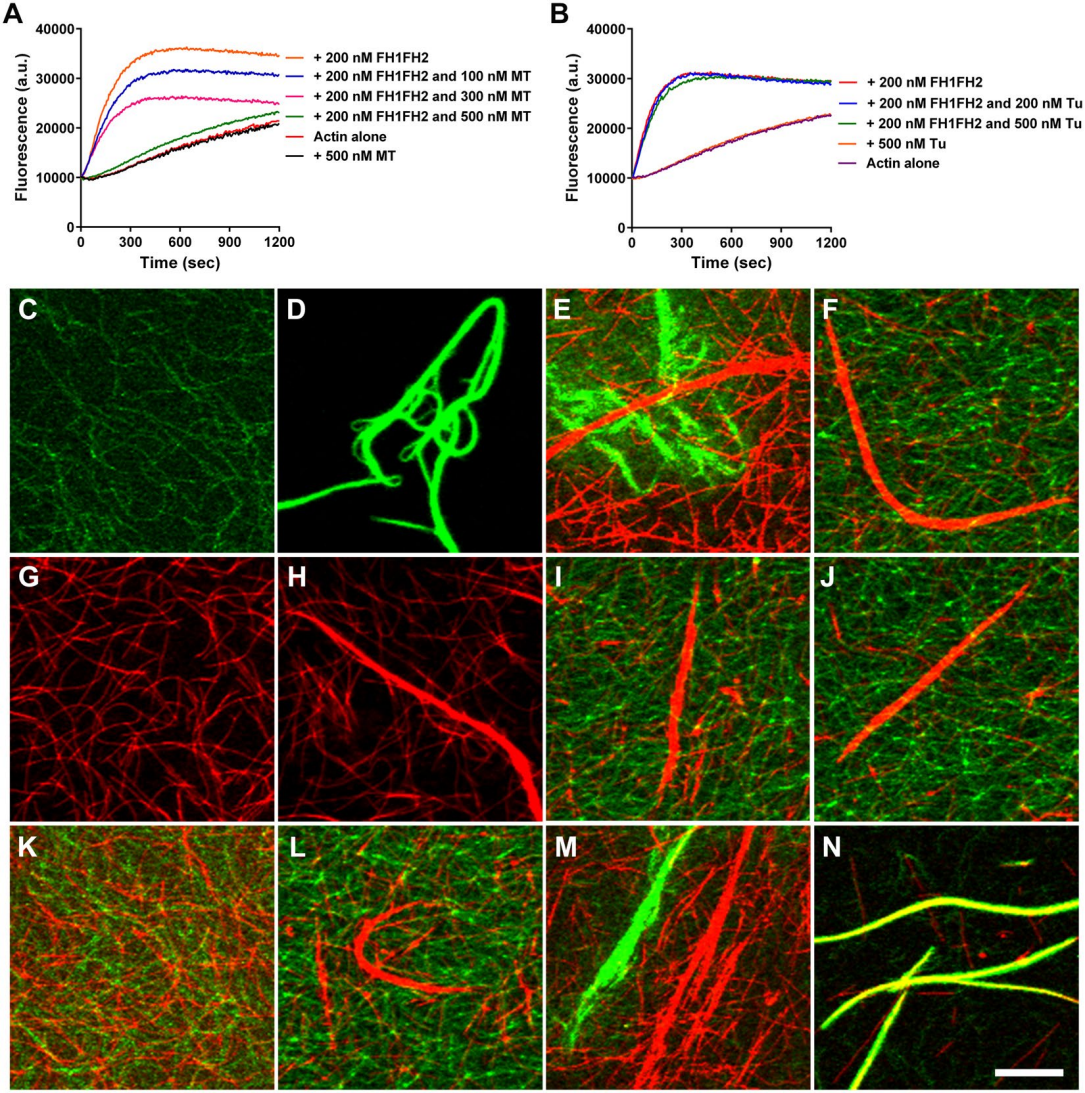
FH1FH2 Preferentially Binds MTs and Crosslinks AFs and MTs *in Vitro*. (A) Time course of actin polymerization in the presence of FH1FH2 and MTs monitored by pyrene fluorescence. Different concentrations of taxol-stabilized MTs were incubated with 200 nM FH1FH2 and 2 μM actin (10% pyrene labeled) before initiation of polymerization. a.u., arbitrary units. (**B**) Time course of actin polymerization in the presence of FH1FH2 and tubulin monomers monitored by pyrene fluorescence. Different concentrations of tubulin monomers were incubated with 200 nM FH1FH2 and 2 μM actin (10% pyrene labeled) before initiation of polymerization. a.u., arbitrary units. (**C**) Preformed AFs labeled with Alexa-488 phalloidin without FH1FH2. (**D**) AFs exhibited considerable bundles 30 min after addition of FH1FH2. (**E**) and (**F**) Addition of preformed taxol-stabilized rhodamine-conjugated MTs to the reaction in (**D**) resulted in the formation of MT bundles and AF bundles decrease within 30 min (**E**) and 4 h (**F**). (**G**) Taxol-stabilized rhodamine-conjugated MTs without FH1FH2. (**H**) MTs exhibited considerable bundles 30 min after addition of FH1FH2. (**I**) and (**J**) MTs remained bundled and AFs were randomly scattered throughout the solution 30 min (**I**) and 4 h (**J**) after the addition of preformed AFs to the reaction in (**H**). (**K**) AFs and MTs were scattered randomly throughout the suspension without FH1FH2. (**L**) MT bundles formed at 30 min after the addition of low concentration FH1FH2 (500 nM) to the reaction in (**K**), but AF bundles were not formed. (**M**) and (**N**) Excess FH1FH2 proteins (2 μM) induced bundling of both MTs and AFs at 30 min (**M**) and crosslinking bundles of the MTs and AFs at 4 h (**N**) after addition. Bar in (**L**) = 10 μm.

Past researches indicated that interactions between formin and one kind of cytoskeleton can be affected by the other kind, for example, actin monomer inhibits MT binding and bundling by INverted Formin 2 (INF2), whereas MTs inhibit actin nucleation by mouse diaphanous-related formin 2 (mDia2)^40, 41^. Given that OsFH15 can bind and bundle both AFs and MTs, we determined the behavior of OsFH15 in the presence of both AFs and MTs using pyrene-actin assays. As shown in Fig. 7A, taxol-stabilized MTs increased the initial lag phase of FH1FH2 nucleating actin polymerization in a concentration-dependent manner. By contrast, tubulin monomers presented no effects on the ability of FH1FH2 in nucleating actin assembly (Fig. 7B). These results indicated that MTs affected the interaction of FH1FH2 with actin. We used fluorescence microscopy to directly visualize effects of FH1FH2. Experiments were performed using 1 µM taxol-stabilized rhodamine-conjugated MTs, 1 µM Alexa-488 phalloidin labeled AFs and 500 nM FH1FH2. FH1FH2 assembled individual AFs (Fig. 7C) into actin bundles (Fig. 7D), and MT bundles formed within 30 min after preformed taxol-stabilized MTs were added to reactions containing actin bundles and FH1FH2, simultaneously, some actin bundles loosened (Fig. 7E). After 4 h incubation, more MT bundles were observed, and actin bundles completely disassembled into individual AFs (Fig. 7F). These results indicated that MTs induced detachment of FH1FH2 from actin bundles. In contrast to disassembly of actin bundles, when preformed AFs were added to reactions containing MT bundles and FH1FH2 (Fig. 7H), MT bundles remained intact, and actin bundles did not form after 30 min or 4 h incubation (Fig. 7I,J). These observations suggested that AFs cannot strip FH1FH2 from MT bundles. Without FH1FH2, AFs and MTs remained as individual filaments that were scattered randomly throughout the solution (Fig. 7K). However, addition of FH1FH2 induced MT bundling, but no actin bundles were observed after 4 h incubation (Fig. 7L). These results indicated that FH1FH2 preferentially bound MTs over AFs. When preformed AFs and MTs were mixed with high concentrations of FH1FH2 (2 µM), both AFs and MTs not only formed bundles (Fig. 7M) but also co-localized (Fig. 7N), these phenomena was also observed under high concentrations of FH2 (Supplemental Fig. 9I).

## Discussion

Grain size and weight are two important indicators of rice quality, and the biological mechanism by which grain shape is regulated is complicated. Previous researches on QTL mapping and cloning have made significant progress on identification of the genes, such as GW8, GL7 and GSK2, regulating the grain size and weight^3^. These genes were mostly targeted for domestication, and grain size and yield were improved by increasing cell size and/or cell number of hulls. An increasing body of evidence showed that cell size is related to the cytoskeleton. In human Wilms’ tumors, the therapeutic agent all-*trans* retinoic acid induces the pronounced increase in cell size with formation of strong actin fibers, as observed from phalloidin staining^42^. In fission yeast, overexpression of the β-tubulin-like cDNA (*Gh*-*BTubL*) from cotton, sharing high sequence identity with plant and yeast β-tubulin, induces longitudinal elongation of the host cells^43^. Although cytoskeleton is related to cell size, no research discussed mechanisms of cell size regulation by cytoskeleton in rice spikelet hulls. In this study, we discovered that knock-out or knock-down of *OsFH15* induced decrease of cell size with reduced amounts of AFs and MTs in lemma and anther (Figs 2,3 and Supplemental Figs 3,5). By contrast, *OsFH15*-overexpressing plants showed increase of cell size with more abundant AFs and MTs in lemma and anther (Figs 2,3 and Supplemental Figs 3,5). These results suggest that OsFH15 can interact with both AFs and MTs to control grain size by regulation of cell area in rice. It is reported that overexpression of *PwTUA1*, a tubulin gene from *Picea wilsonii*, induces more α-tubulin and vesicles accumulating in the apex region of pollen tube compared with wild-type *Arabidopsis*, overexpressing *PwTUA1* can increases pollen tube elongation by changing the distribution of α-tubulin and promoting vesicle transport^44^. Moderate overexpression of AtFH5, an *Arabidopsis* actin-nucleating protein, increases subapical actin prominence in pollen tube. These AFs are used as tracks for vesicular transport to increase pollen tube growth rate^45^. Considering that OsFH15 can regulate both AFs and MTs, we propose that more abundant AFs and MTs and/or interaction between AFs and MTs promote cell expansion by promoting vesicles trafficking. However, it is not proportional between higher *OsFH15* mRNA level and organ growth, as spikelet hull of the overexpression transgenic plant produces 150-fold more *OsFH15* mRNA than wild type but the spikelet hull size increases by just 12% (Fig. 1C,D). This premise suggests that there is a physical limitation to the extent of cell expansion in spikelet hull, or that excessive *OsFH15* mRNAs may not translate large amounts of proteins and that the high ectopic expression of *OsFH15* increases local cell expansion to only a minor extent.

In recent years, cooperation of AFs with MTs in plant cells drew major attention. Various articles showed that some proteins can interact with both AFs and MTs to execute fundamental cellular and developmental processes in plant cells^20, 21, 24, 25, 39^. In this study, OsFH15, identified to the first one in rice, not only binds both AFs and MTs but also crosslinks these cytoskeletons (Figs 6,7 and Supplemental Figs 9,10). AFs and MTs, are mediated by specific bifunctional proteins or multiprotein complexes and often act physically in a coordinated manner^16^. In this report, OsFH15 interacted with both AFs and MTs (Figs 6,7 and Supplemental Figs 9,10), indicating that OsFH15 may be a potential bifunctional protein for these two cytoskeletal components in living plant cells. To gain insights into physiological functions of OsFH15, we stained AFs with Alexa-488 phalloidin and immunostained MTs in cells of rice lemma and anther. Results showed that the amount and bundles of both AFs and MTs reduced in *Osfh15* mutant cells and increased in *OsFH15* OE cells, indicating that OsFH15 may affect the cooperation of AFs and MTs (Fig. 3, Supplemental Fig. 5). Besides OsFH15 binding or crosslinking AFs and MTs, we noted that OsFH15 also sufficiently bundles AFs or MTs (Fig. 6, Supplemental Figs 9,10), demonstrating that OsFH15 may function by stabilizing AFs and MTs. Universal bundles of AFs and MTs reduced in *Osfh15* mutant cells and increased in *OsFH15* OE cells (Fig. 3, Supplemental Fig. 5), further indicating that OsFH15 plays a prominent role in stabilizing AFs and MTs *in vivo*. Previous studies showed that formins can associate with actin and are key regulators of actin nucleation and elongation in eukaryotes. Many researches showed that most reported plant formins can nucleate actin polymerization via the FH1FH2 domain^46^, except for AtFH16^24^. OsFH15 corresponds to a class I formin^32, 33^ and features a characteristic polyproline-stretch FH1 domain and a typical FH2 domain (Supplemental Fig. 7A). Presence of these two typical domains implies that OsFH15 may retain all general functions of formins. Indeed, OsFH15 FH1FH2 is able to nucleate actin assembly (Fig. 4). However, nucleation activity of OsFH15 FH2 was not detected (Supplemental Fig. 8). Results suggest that the FH1 domain is necessary for OsFH15 to nucleate actin assembly. In some plant cells, the majority of actin is sequestered by equimolar profilin^34, 47^. OsFH15 promotes actin assembly in the presence of OsPRF1 or OsPRF2 (Fig. 4), implying that OsFH15 can be a positive actin-nucleator *in vivo*. Consistent with this hypothesis, AF level decreased in *Osfh15* mutant cells and increased in *OsFH15* OE cells (Fig. 3, Supplemental Fig. 5). Moreover, OsFH15 exhibits barbed-end capping activity (Fig. 5), which suggests that OsFH15 may function in stabilizing AFs.

In flowering plants, seeds comprise the embryo, endosperm and seed coat. Endosperms can occupy approximately 80% of seed mass in rice^48^. Control of endosperm growth largely determines final sizes of seeds in cereals^49^. In this study, *OsFH15* transcripts were abundant in seeds. Moreover, the expression level of *OsFH15* gradually increased with the development of seeds (Fig. 1A), indicating that OsFH15 may be involved in regulating endosperm cell size to control grain size, this topic requires further research. The mechanism of OsFH15 regulating tillers and plant height also need further study.

In conclusion, we provide genetic and biochemical evidence to demonstrate that OsFH15 regulates rice grain size by affecting cell expansion through its influence over AFs and MTs or by possibly controlling the interactions between AFs and MTs. This work provides new insights into the developmental and physiological roles of class I formins in higher plants.

## Methods

### Mutant Materials and Growth Conditions

The rice (*Oryza sativa*) *OsFH15* mutant was isolated from CRISPR/Cas9-mediated gene editing-induced mutations of a *japonica cultivar* (Hwayoung). Plants were cultivated in an experimental field of Beijing Normal University during natural growing seasons. Seedlings were germinated and grown on Murashige and Skoog (MS) medium in a growth chamber under growth conditions with 12 h light 30 °C and 12 h dark 28 °C at 60% relative humidity.

### Observation and Quantification of AFs and MTs in Plant Cells

The actin staining procedure in rice lemma has been described previously by Yang *et al*.^39^. For data collection, AFs were observed by an LSM510 laser scanning confocal microscope (Zeiss, http://www.zeiss.com). The optical Z-series step size was set at 0.5 μm. And there are 4–5 Z sections in different samples. The pinhole size of 2 Airy units was employed for imaging. Alexa-488 phalloidin was excited with a 488 nm blue argon laser, and the emission wavelength was set at 550–600 nm. Immunostaining of MTs was performed on the plant cells according to previously published methods^39^. Images were prepared by generating projections of the optical sections through an individual epidermal cell which was from rice lemma. Average fluorescence density of AFs and MTs were quantified using a set of previously validated tools^50^. The average pixel fluorescence intensity was obtained with ImageJ, then was plotted and analyzed in Microsoft Office Excel.

### qRT-PCR Analysis

SAM was taken from the 30 d-old seedling. The outer leaves were carefully stripped under stereoscope microscope until SAM was discovered. SAM was isolated by using the surgical blade and quickly frozen in liquid nitrogen for further experiments. Spikelets, spikelet hulls and seeds were collected in different developmental stage, and total RNA was isolated from roots, stems, leaves, SAM, Spikelets, spikelet hulls and seeds using Trizol reagent (Tiangen) according to the manufacturer’s instructions. The cDNA was prepared by reverse transcription with transscript reverse transcriptase (Transgen). We amplified partial coding regions of *OsFH15* with primer pair OsFH15-QRT-F1/OsFH15-QRT-R1 (Supplemental Table S1) to determine the transcript levels of *OsFH15*, and the rice *UBQ5* was used as the control gene using primer pair UBQ5-QRT-L/ UBQ5-QRT-R (Supplemental Table S1).

The qRT-PCR analysis was performed using iQ SYBR Green Supermix (Bio-Rad) on the iQ5 multicolor real-time PCR detection system (Bio-Rad). The total volume of 20 μL reaction mixture contain 10 μL iQ SYBR Green Supermix, 300 nM each primer and 100 ng reverse transcription products were used as template. The qRT-PCR procedures were performed for each sample according to the manufacturer’s protocol (Bio-Rad). The samples were denatured for 3 min at 95 °C; and then followed by 40 cycles of 15 s of denaturation at 95 °C, 30 s of annealing at 58 °C, 30 s of elongation at 72 °C; followed by one cycle of 5 min of elongation at 72 °C; followed lastly by 61 cycles of 15 s of annealing at 65 °C. Each experiment was carried out in three biological replicates. The relative expression levels were analyzed using the comparative C_T_ method.

### Protein Production

To generate recombinant FH1FH2 and FH2, these recombinant proteins were amplified with primer pairs OsFH15-FF-L1/OsFH15-FF-R1 and OsFH15-F-L1/OsFH15-F-R1, respectively, and cloned in frame with 6 × His in pET-30a(+) vector, respectively. Then, the two constructs were transformed into the Transetta (DE3) strain of *Escherichia coli*. Cells were grown to an OD_600 _=0.6 at 37 °C and treated with 0.5 mM isopropylthio-β-galactoside at 20 °C overnight to induce recombinant proteins expression. Cultures were collected by centrifugation at 5000 g for 10 min at 4 °C and resuspended in binding buffer (400 mM NaCl and 40 mM PBS, pH 8.0). The recombinant proteins were purified using a Ni-NTA affinity column following the protocol in the manufacturer’s instructions (Novagen). The column was washed with washing buffer (2 mM 2-mercaptoethanol, 20 mM imidazole, 250 mM KCl, and 25 mM Tris-HCl, pH 7.9), then, the recombinant proteins were eluted with eluting buffer (2 mM 2-mercaptoethanol, 40 mM imidazole, 250 mM KCl, and 25 mM Tris-HCl, pH 7.9) and dialyzed overnight against TK buffer (0.5 mM DTT, 0.5 mM EDTA, 5 mM Tris, and 50 mM KCl). The purified protein concentrations were determined with the Bradford assay (Bio-Rad) using BSA as a standard.

OsPRF1 and OsPRF2 were purified with poly-L-proline Sepharose. OsPRF1 and OsPRF2 were expressed in *Escherichia coli*. BL21 (DE3) using a pET-30a(+) plasmid. Briefly, the bacteria were transformed with the plasmids and grown in 50 mg/L kanamycin at 37 °C. At OD_600_ = 0.5, protein expression was induced at 18 °C with 0.6 mM isopropyl β -D-thiogalactoside. After 16 h inducement in LB media, bacteria cells were harvested by centrifugation. The pellet was resuspended in buffer I (150 mM KCl, 20 mM Tris, 0.2 mM DTT, pH 7.5) and broken by sonication. The sonicate was subsequently centrifuged at 40,000 *g* for 30 min. Then, profilins were purified by affinity chromatography on poly-L-proline Sepharose as described previously^51, 52^. After adsorbing the sample, the column was washed with three volumes of buffer I, with an additional wash of buffer I plus 3 M urea before the elution with buffer I plus 7 M urea. The protein-containing fractions were pooled and dialyzed against buffer I, aliquoted, and stored in the liquid nitrogen for future use.

### Preparation and Polymerization of Actin and Tubulin

Actin was purified from rabbit muscle acetone powder using one cycle of polymerization and depolymerization followed by gel filtration and storage in Ca-Buffer-G (2 mM Tris, 2 mM ATP, 0.1 mM CaCl_2_, 1 mM NaN_3_, 0.5 mM DTT, pH 8.0)^53, 54^. For Oregon-green labeling, gel-filtered actin in 25 mM imidazole (pH 7.5), 100 mM KCl, 2 mM MgCl_2_, 3 mM NaN_3_, and 0.3 mM ATP was labeled on Cys374 by being incubated overnight at 4 °C with a 1: 12 molar ratio of actin to Oregon-green iodoacetamide^55^. For pyrene iodoacetamide labeling, gel-filtered actin in 25 mM Tris (pH 7.5), 100 mM KCl, 2 mM MgSO_4_, 3 mM NaN_3_ and 0.3 mM ATP was labeled on Cys374 by being incubated overnight at 4 °C with a 1: 7 molar ratio of actin to pyrene iodoacetamide^55, 56^. Ca-ATP-actin was converted to Mg-ATP-actin by addition of 0.1 volume of 2 mM EGTA and 500 μM MgCl_2_ and incubation of the sample for 5 min at room temperature^57^.

Porcine brain tubulins were purified according to a previously published method^58^. The 5-(and 6-) carboxytetramethylrhodamine succinimidyl ester (NHS)-rhodamine was then conjugated to purified tubulin as previously reported^59, 60^. NHS-rhodamine-labeled tubulin then underwent another round of assembly/disassembly with 10% (v/v) dimethyl sulfoxide before being stored in liquid nitrogen as 10 μL aliquots, which were later used for polymerization and sedimentation assays.

Actin was centrifuged at 100,000 g for 1 h using a TLA-110 rotor (Beckman) at 4 °C to remove denatured actin. Actin (10 μM) was polymerized in 10 × KMEI buffer at 4 °C overnight or at 37 °C for 2 h in the presence of 100 nM Alexa-488 phalloidin. To polymerize the tubulin into MTs, tubulin or NHS-rhodamine-labelled tubulin were centrifuged at 50, 000 g for 30 min at 4 °C before polymerization. Tubulin or NHS-rhodamine-labelled tubulin were diluted with PEM buffer (0.1 M PIPES, 1 mM EGTA, 1 mM MgSO_4_, pH 6.9) containing 1 mM GTP (guanosine 5′-triphosphate sodium salt hydrate) to a concentration of 40 μM, and incubated at 37 °C for 30 min. Two volumes (v/v) of PEMT (0.1 M PIPES, 1 mM EGTA, 1 mM MgSO_4_, 20 mM taxol, pH 6.9) were added to the solution, which was incubated at 37 °C for 15 min and centrifuged at 12, 000 g at 25 °C for 15 min. The pellets were washed with PEMT and resuspended gently with PEMT to give a final concentration of 10 μM tubulin.

### AF Elongation and AF Depolymerization Assay

The seeded AF elongation assay was performed according to the method previously described^38^. Freshly performed AF seeds (0.8 μM) were incubated with different concentrations of FH1FH2 for 5 min at room temperature. Actin elongation at the barbed end of AFs was initiated by adding 1 μM G-actin (10% pyrene-labeled) and one-tenth volume of 10× KMEI buffer. The change in pyrene fluorescence accompanying actin polymerization was monitored after actin elongation was initiated.

Dilution assays were performed as described by by Zhang *et al*.^25^. Preformed AFs (5 μM, 60% pyrene-labeled) was incubated with different concentrations of FH1FH2 at room temperature for 5 min, before the solution was diluted 25-fold into G buffer (2 mM Tris-HCl, pH 8.0, 0.1 mM CaCl_2_, 0.2 mM ATP, and 0.5 mM DTT). The decrease of pyrene fluorescence intensity accompanying actin depolymerization was monitored after dilution.

Methods S1–S6 can be found in Supplemental Information.

### Data Availability

Sequence data from this article can be found in the GenBank databases under the following accession numbers: Os10g17660 for OsPRF1, and Os06g05880 for OsPRF2.

## Electronic supplementary material

FH1FH2 Caps the Barbed Ends of AFs and Blocks the Elongation.

FH1FH2 can Accelerate AFs Elongation in the Presence of OsPRF1.

FH1FH2 Induced AFs into Bundles.

Supplementary Information
