# Supplementary material for: OsFH15, a class I formin, interacts with microfilaments and microtubules to regulate grain size via affecting cell expansion in rice
Source: Sci Rep. 2017 Jul 26;7:6538. doi: 10.1038/s41598-017-06431-5 (PMC5529588; doi:10.1038/s41598-017-06431-5)
Supplement: Supplementary file 4 — Supplementary Information [file 41598_2017_6431_MOESM4_ESM.pdf]

**Supplemental Information for**

# OsFH15, a class Ⅰ formin, interacts with microfilaments and microtubules to regulate grain size via affecting cell expansion in rice

Tiantian Sun†, Shanwei Li† and Haiyun Ren*

Key Laboratory of Cell Proliferation and Regulation Biology of Ministry of Education, College of Life Science, Beijing Normal University, Beijing 100875, China

† These authors contributed equally to this work.

* Author for correspondence:

Haiyun Ren

Tel: +86 10 58806090

Email: hren@bnu.edu.cn

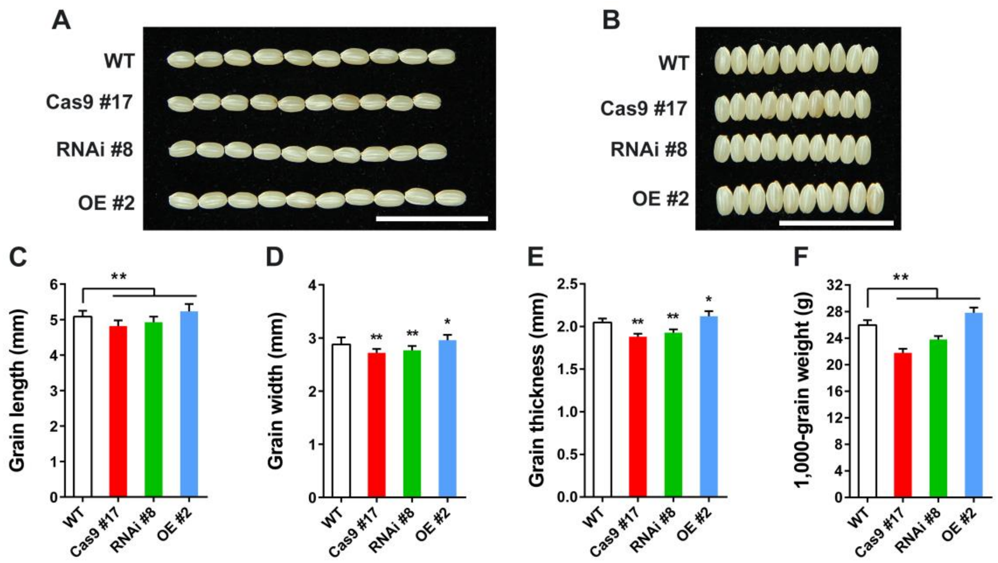


**Supplemental Figure 1. Phenotype with Overexpression and Repression of *OsFH15*.**

(A) and (B) Grains of WT, Cas9 #17, RNAi #8 and OE #2. Bar in (A) and (B) = 2 cm.

(C) to (F) Grain length (C), Grain width (D) and Grain thickness (E) were determined by vernier depth. 1,000-grain weight (F) was weighed. Data are represented as mean ± SEM (n ≥ 100 in C-E, n = 15 in F). *P < 0.05, **P < 0.01, Student's *t* tests were used to generate *P* values.

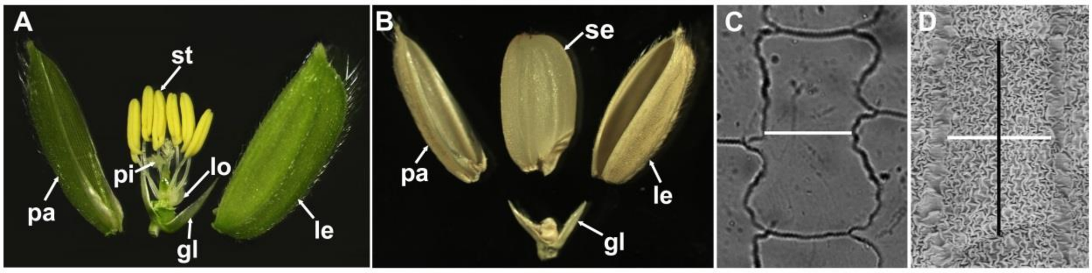


**Supplemental Figure 2. The Model of a Spikelet and Its Cells.**

(A) The model of a spikelet. Le, lemma; pa, palea; pi, pistil; st, stamen; lo, lodicule; gl, glume. The spikelet hull contains lemma and palea.

(B) The model of a mature rice grain. Le, lemma; pa, palea; gl, glume; se,seed. The spikelet hull contains lemma and palea.

(C) The model of an inner epidermal cell of lemma.

(D) The model of an epidermal cell of anther.

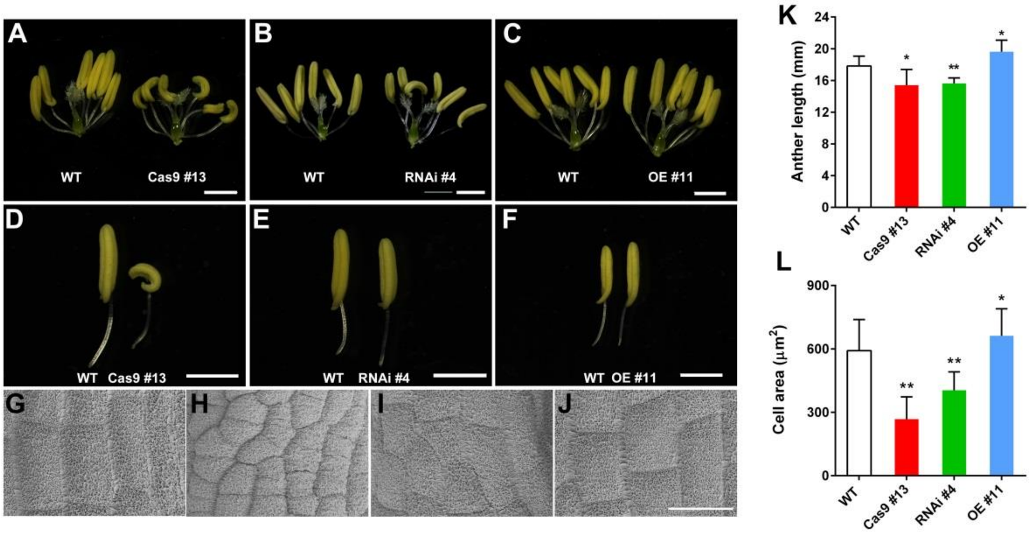


**Supplemental Figure 3. Floral Organs with Overexpression and Repression of *OsFH15*.**

(A) to (C) Pistils and stamens before anthesis comparison between wild type and Cas9 #13 (A), RNAi #4 (B), OE #11 (C), respectively.

(D) to (F) Stamens before anthesis comparison between wild type and Cas9 #13 (D), RNAi #4 (E), OE #11 (F), respectively.

(G) to (J) Cells on short locules of anthers in the wild type (G), Cas9 #13 (H), RNAi #4 (I), OE #11 (J) by scanning electron microscopy.

(K) to (L) Statistical data of the anther length (K) and the cell area of short locules of anthers (L). Bar in (A) to (F) = 2 mm, Bar in (J) = 30 μm. Data are represented as mean ± SEM (n ≥ 50 in K, n ≥ 100 in L). *P < 0.05, **P < 0.01, Student's *t* tests were used to generate *P* values.

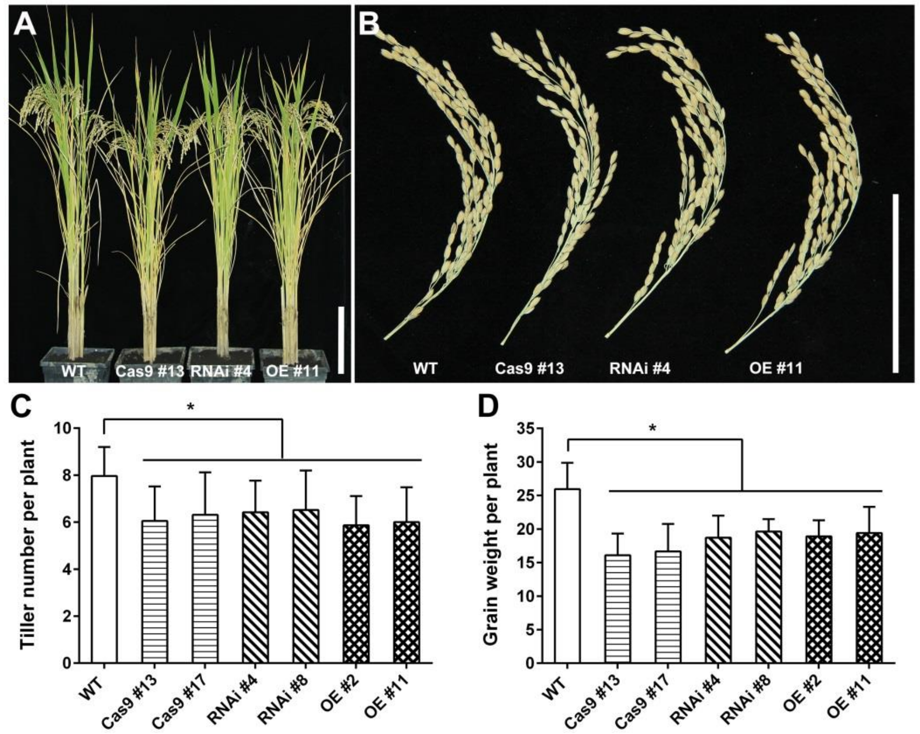


**Supplemental Figure 4. Morphological Phenotype with Overexpression and Repression of *OsFH15*.**

(A) Morphology of mature plants, Bar = 18 cm.

(B) The panicles, Bar = 9 cm.

(C) Tiller number per plant at the stage showing in (A).

(D) Grain weight per plant were weighted. Data are represented as mean ± SEM (n ≥ 50 in C and D). *P < 0.05, Student's *t* tests were used to generate *P* values.

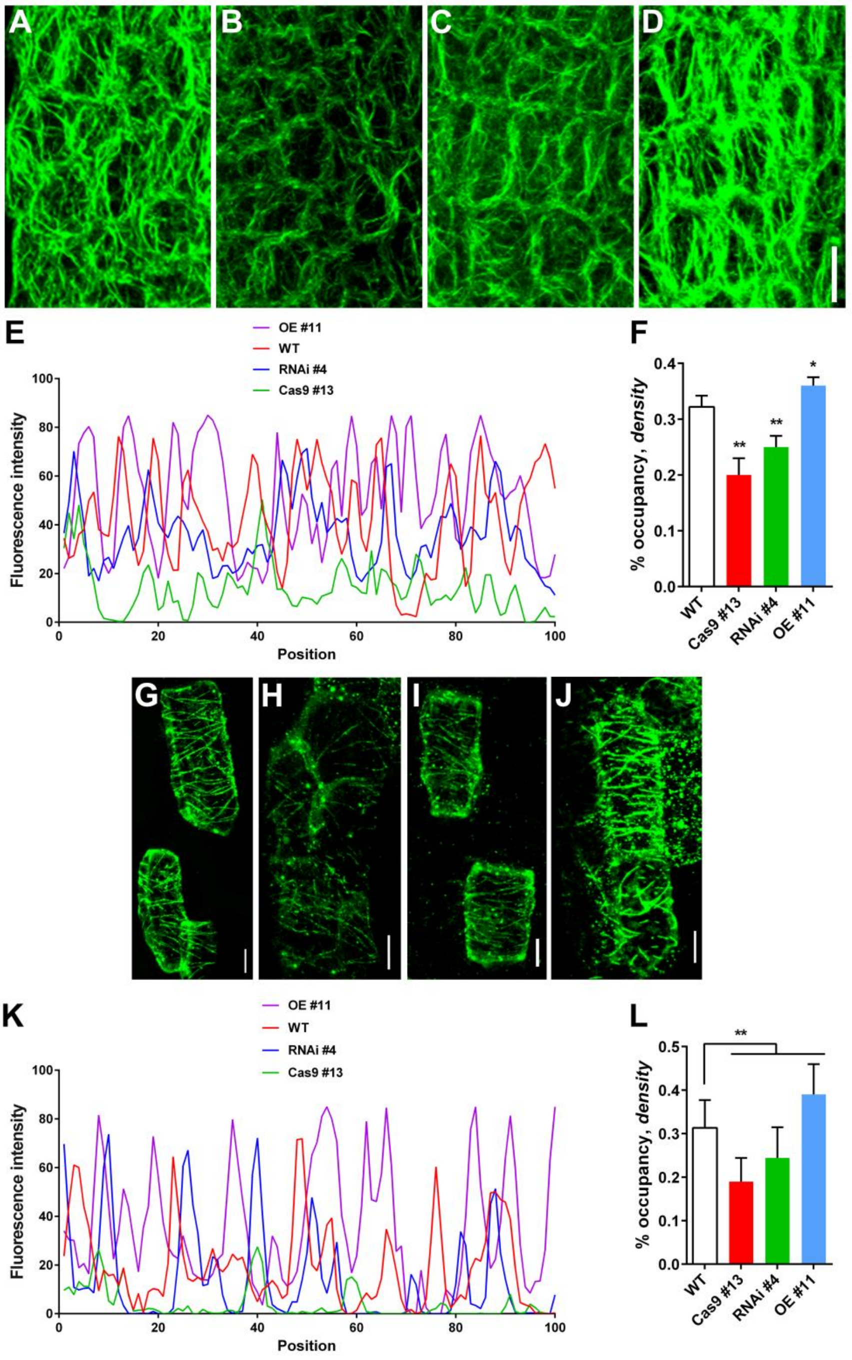


**Supplemental Figure 5. AF and MT Organization in Wild-Type and Transgenic Cells of Anthers.**

(A) to (D) AF organization was stained by Alexa Fluor 488-phalloidin, WT (A), Cas9 #13 (B), RNAi #4 (C), OE #11 (D).

(G) to (J) MTs were stained with tubulin antibody, WT (G), Cas9 #13 (H), RNAi #4 (I), OE #11 (J). (A) to (D) and (G) to (J) The cells from the epidermal cells of anthers.

(E) Fluorescence intensities corresponding to the regions of cell middle part in (A) to (D), this cell middle part was marked in Supplemental Fig. 2D with white line.

(K) Fluorescence intensities corresponding to the regions of cell middle part in (G) to (J), this cell middle part was marked in Supplemental Fig. 2D with black line.

(F) and (L) Average AF and MT density were measured and binned for the same regions in (A) to (D) and (G) to (J), respectively. Bar in (D) and (G) to (J) = 10 μm. Data are represented as mean ± SEM (n ≥ 50 in F and L). *P < 0.05, **P < 0.01, Student's *t* tests were used to generate *P* values.

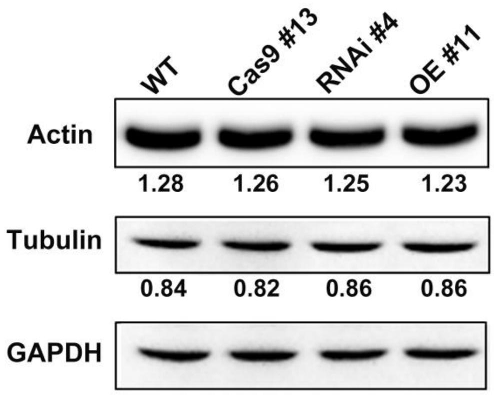


**Supplemental Figure 6. The Amount of Total Actin and Tubulin Does Not Differ in Wild-Type and Transgenic Plants.**

Total proteins were isolated from lemmas of WT, Cas9 #13, RNAi #4 and OE #11. Protein blots were incubated with an anti-actin antibody, an anti-tubulin antibody and an anti-GAPDH antibody. GAPDH was used as internal controls. Levels of total actin and tubulin were calculated against the level of total GAPDH in each sample.

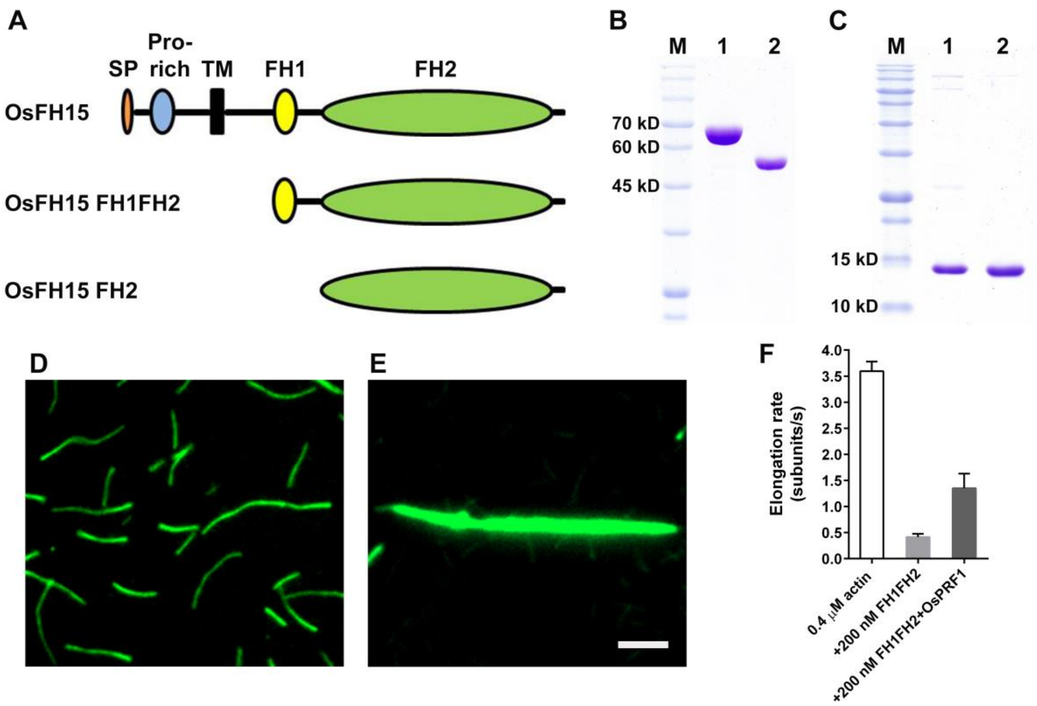


**Supplemental Figure 7. The Bundling and Elongation of AFs in the Presence of FH1FH2 and/or OsPRF1.**

(A) Schematic representation of the predicted domain organization of OsFH15. OsFH15 contains a signal peptide (SP, a.a. 1-18), Pro-rich (a.a. 44-86), a transmembrane domain (TM, a.a. 148-168), an FH1 domain (FH1, a.a. 257-297) and an FH2 domain (FH2, a.a. 340-768). Recombinant FH1FH2 and FH2 fusion proteins include a.a. 257-768 and 340-768, respectively.

(B) Coomassie-stained 10% SDS-PAGE of purified recombinant OsFH15 proteins after purification. Lane M, molecular weight markers in kDa; lane 1, FH1FH2 protein; lane 2, FH2 protein.

(C) Coomassie-stained 15% SDS-PAGE of purified two rice profilin proteins after purification with poly-L-proline Sepharose. Lane M, molecular weight markers in kDa; lane 1, OsPRF1 protein; lane 2, OsPRF2 protein.

(D) Preformed AFs labeled with Alexa-488 phalloidin in the presence of OsPRF1.

(E) AFs formed considerable bundles after addition of FH1FH2 in the presence of OsPRF1. Bar = 5 μm.

(F) Average elongation rates of actin alone (0.4 μM, 30% Oregon-green labeled) and FH1FH2-assembled AFs in the absence and presence of OsPRF1 (2.0 μM). Data are

represented as mean ± SEM, n ≥ 30, N = 3.

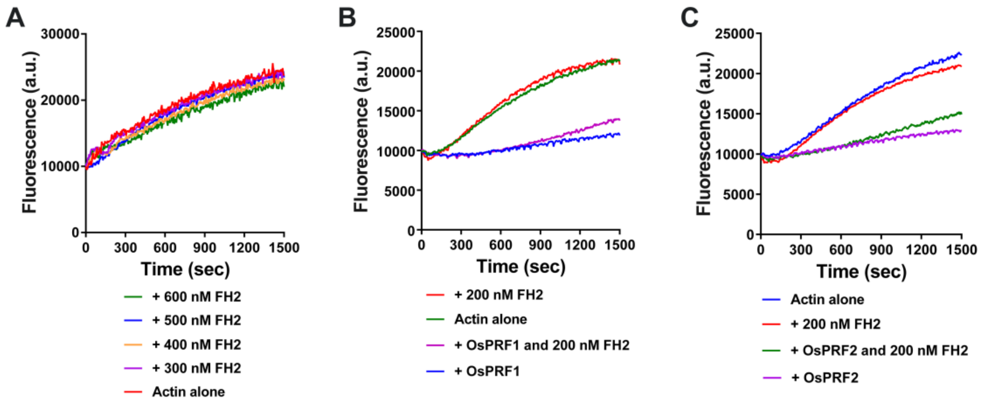


**Supplemental Figure 8. FH2 Does Not Nucleate Actin Polymerization with or without Profilin.**

(A) Time course of actin polymerization in the presence of FH2 monitored by pyrene fluorescence. Different concentrations of FH2 were added to 2 μM actin (10% pyrene labeled) before initiation of polymerization. a.u., arbitrary units.

(B) Time course of actin polymerization in the presence of FH2 and/or OsPRF1.

(C) Time course of actin polymerization in the presence of FH2 and/or OsPRF2.

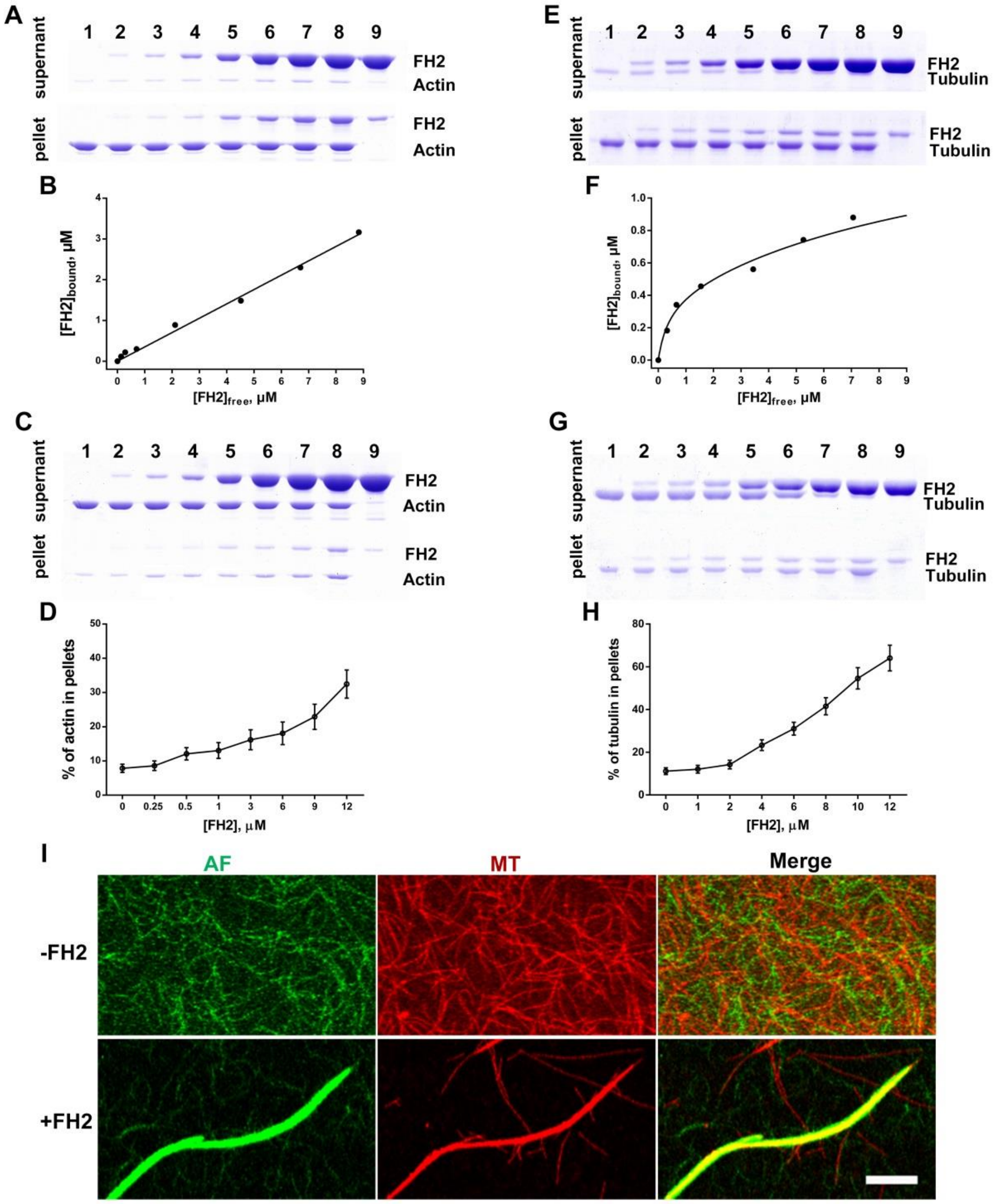


**Supplemental Figure 9. FH2 Binds, Bundles and Links AFs and MTs *in Vitro*.**

(A) A high-speed cosedimentation assay was performed to assess the binding between FH2 and AFs. Lane 1, actin alone (3 μM); lanes 2-8, actin plus 0.25, 0.5, 1, 3, 6, 9, or 12 μM FH2, respectively; Lane 9, FH2 alone (12 μM).

(B) Quantification of scanned SDS-PAGE gel from (A). The concentration of bound FH2 was plotted against the concentration of free FH2 and fitted with a hyperbolic function. For this representative experiment, the *Kd* was calculated to be 1.51 μM.

(C) A low-speed cosedimentation assay was performed to assess the bundling activity between FH2 and AFs. Lane 1, actin alone (3 μM); lanes 2-8, actin plus 0.25, 0.5, 1, 3,

6, 9, or 12 μM FH2, respectively; Lane 9, FH2 alone (12 μM).

(D) Percentage of AFs recovered in the low-speed pellet in different concentration of FH2.

(E) A high-speed cosedimentation assay was used to determine the binding affinity between FH2 and MTs. Lane 1, tubulin alone (1 μM); lanes 2-8, tubulin plus 0.5, 1, 2, 4, 6, 8, or 10 μM FH2, respectively; Lane 9, FH2 alone (10 μM).

(F) Quantification of scanned SDS-PAGE gel from (E). The concentration of bound FH2 was plotted against the concentration of free FH2 and fitted with a hyperbolic function. For this representative experiment, the *Kd* was calculated to be 1.81 μM.

(G) A low-speed cosedimentation assay was performed to assess the bundling activity between FH2 and MTs. Lane 1, tubulin alone (1 μM); lanes 2-8, tubulin plus 1, 2, 4, 6, 8, 10 or 12 μM FH2, respectively; Lane 9,FH2 alone (12 μM).

(H) Percentage of MTs recovered in the low-speed pellet in different concentration of FH2.

(I) Micrographs of AFs (0.5 μM) or MTs (0.5 μM) in the absence or presence of FH2. FH2 (3 μM) induced AFs into bundles (left lane) and MTs into bundles (middle lane), even crosslinking the bundles of the AFs and MTs (right lane). Bar = 10 μm.

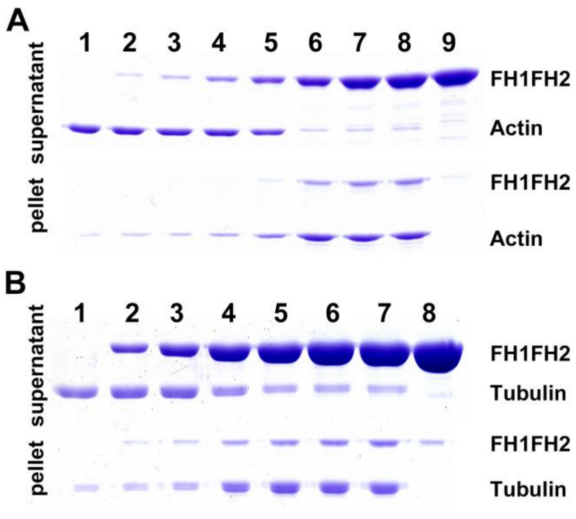


**Supplemental Figure 10. FH1FH2 Bundles AFs and MTs *in Vitro*.**

(A) A low-speed cosedimentation assay was performed to assess the bundling activity between FH1FH2 and AFs. Lane 1, actin alone (3 μM); lanes 2-8, actin plus 0.25, 0.5, 1, 2, 4, 6, or 8 μM FH1FH2, respectively; Lane 9, FH1FH2 alone (8 μM).

(B) A low-speed cosedimentation assay was performed to assess the bundling activity between FH1FH2 and MTs. Lane 1, tubulin alone (1 μM); lanes 2-7, tubulin plus 1, 2, 4, 6, 8, or 10 μM FH1FH2, respectively; Lane 8, FH1FH2 alone (10 μM).

**Supplemental Movie 1. FH1FH2 Caps the Barbed Ends of AFs and Blocks the Elongation.**

Time-lapse of actin (0.4 μM, 30% Oregon-green labeled) polymerization in the different concentration of FH1FH2.

**Supplemental Movie 2. FH1FH2 can Accelerate AFs Elongation in the Presence of OsPRF1.**

Spontaneous assembly of actin (0.4 μM, 30% Oregon-green labeled) or the assembly of actin (0.4 μM, 30% Oregon-green labeled) in the presence of 200 nm FH1FH2 or 200 nm FH1FH2 with 2.0 μM OsPRF1 were visualized by the TIRFM.

**Supplemental Movie 3. FH1FH2 Induced AFs into Bundles.**

Time-lapse micrographs of the assembly of actin (0.7 μM, 15% Oregon-green labeled) with FH1FH2 (right) or without FH1FH2 (left) were visualized by the TIRFM.

**Table S1. Primers Used for This Study**

| Primer Name | Primer sequences (5’ → 3’) |
|---|---|
| 15-1-F | AGCATCCAAGAATGGAGTCAAGG |
| 15-1-R | CCTTGACTCCATTCTTGGATGCT |
| OsFH15-802iF | AAGGTACCACTAGTAGATGGTGGTACGGACGCAG |
| OsFH15-802iR | AAGGATCCGAGCTCCACCTTGTCCCAGTGCAGCG |
| OsFH15-407iF | AAGGTACCACTAGTCGAACATCTCGTACATGGCC |
| OsFH15-407iR | AAGGATCCGAGCTCCGCTCGCTTGTCCTCTGCT |
| OsFH15F-1 | AAGGTACCATGCTCGCGCGGTGGCTGCT |
| OsFH15R-1 | AAACTAGTCTAGGACGATGAGCTCTCCTCCT |
| OsFH15-FF-L1 | AACATATGCAAACCATCCATGAAGCC |
| OsFH15-FF-R1 | AAGGATCCTGGTTGAACCTCGCAAGAAC |
| OsFH15-F-L1 | AACATATGGAGGAGAAGGCC |
| OsFH15-F-R1 | AAGGATCCTGGTTGAACCTCGCAAGAAC |
| UBQ5-QRT-L | ACCACTTCGACCGCCACTACT |
| UBQ5-QRT-R | ACGCCTAAGCCTGCTGGTT |
| OsFH15-QRT-F1 | CATTTGCGTTCAAGAGGGTC |
| OsFH15-QRT-R1 | CCAGTGTTCATGCGGTTACC |
| OsPRF1-F | AACATATGATGTCGTGGCAGACGTAC |
| OsPRF1-R | AAGAGCTCCTACAGGCCCTGCTCTAC |
| OsPRF2-F | AACATATGATGTCGTGGCAGGCGTAC |
| OsPRF2-R | AAGAGCTCTTAGCAACCCTGCTCGATC |

Underlines denote introduced restriction sites.

**Methods S1 Constructs and Transformation**

The targeter website (http://cbi.hzau.edu.cn/cgi-bin/CRISPR) was used to identify potential target sites for our RNA-guided Cas9 system. The target sites are 5′-$(N)_{20}$-NGG-3′, and the targeter website will analyze these query sequences and return sites that either flank the nucleotide of interest, or, are as close to it as possible. We obtained a target site: 15-1 AGCATCCAAGAATGGAGTCAAGG. The pP1C.2-15-1 was constructed by annealing of oligonucleotides 15-1, followed by ligation in pP1C.2 (Genloci Biotechnologies Inc.) cut by Bbs Ⅰ. pP1C.2-15-1 was digested by EcoR Ⅰ and Nhe Ⅰ and cloned into pP1C.1 (Genloci Biotechnologies Inc.) which was digested by EcoR Ⅰ and Xba Ⅰ, then we constructed the pCas9 plasmid which was pP1C.1-15-1.

A 2367 bp DNA fragment was amplified from wild-type plants, with primers OsFH15F-1 and OsFH15R-1 (Table S1). The pTCK303 vector has a constitutive maize *Ubi-1* promoter to drive the gene expression. After sequencing, the fragment was inserted into pTCK303 vector digested with BamH Ⅰ/Sac Ⅰ to create *OsFH15* OE plasmids pTCK303-*Ubi*: OsFH15. To construct *OsFH15* RNAi plasmids, an 802 bp gene-specific fragment and a 407 bp gene-specific fragment were amplified using the primer pairs: OsFH15-802iF/OsFH15-802iR and OsFH15-407iF/OsFH15-407iR, respectively (Table S1). These sequences also cloned into the pTCK303 vector to obtain pTCK303-*Ubi*: OsFH15-802i and pTCK303-*Ubi*: OsFH15-407i, respectively.

Above all the plasmids were introduced into rice embryonic calli by *Agrobacterium* transformation.

**Methods S2 Western Blot Analysis**

The lemmas of mature spikelets were collected from WT or *OsFH15* mutant plants. Total proteins of lemmas were subsequently extracted by homogenization in buffer (0.05 M NaF, 0.1 M Tris-HCl, 0.4 M D-Sorbitol, 0.5 M $CaCl_2$, 0.5 mM ATP, 0.5 mM PMSF and 5 mM DTT, pH 7.0) and 10 μg total proteins were analyzed using SDS-PAGE. After SDS-PAGE, proteins on the 12% polyacrylamide gel were electrophoretically transferred to a polyvinylidene difluoride membrane (Millipore),

and the blot was blocked with PBS containing 3% BSA and 0.1% Tween-20 for 1 h. The anti-actin antibody (M20009, Abmart), anti-tubulin antibody (T4026, Sigma) and anti-GAPDH antibody (HC301, Transgen) were used at 1:1000 and 1:5000 dilution, respectively. Proteins of interest were detected with horseradish peroxidase (HRP)-conjugated goat anti-mouse IgG antibody (1:8000; HS201, Transgen). The signals were detected by the enhanced chemiluminescent method.

**Methods S3 Actin Nucleation Assay**

The actin/profilin complex was prepared by adding profilin into actin with a 3 : 1 molar ratio (1 : 3 actin/profilin complex). Mg-ATP-monomeric actin or actin/profilin complex (10% pyrene-labeled) was incubated with different concentrations of recombinant formins at room temperature for 5 min. Pyrene fluorescence was monitored by the addition of one-tenth volume of 10 × KMEI buffer (10 mM EGTA, 10 mM $MgCl_2$, 0.5 mM KCl, 0.1 mM imidazole, pH 7.0) using a fluorescence spectrophotometer F200 PRO (Tecan).

**Methods S4 MT and AF Cosedimentation Assays**

These experimental procedures have been described in previously published article[1] with slight modifications of recombinant proteins concentration. In the low-speed and high-speed cosedimentation assays, the concentration gradient of FH1FH2 is 0, 0.25, 0.5, 1, 2, 4, 6, or 8 μM and FH2 is 0, 0.25, 0.5, 1, 3, 6, 9, or 12 μM in the presence of 3 μM AFs. The concentration gradient of FH1FH2 is 0, 1, 2, 4, 6, 8, 10 or 12 μM and FH2 is 0, 0.5, 1, 2, 4, 6, 8 or 10 μM in the presence of 1 μM MTs.

**Methods S5 Direct Visualization of Actin Assembly and Bundles by TIRFM**

TIRFM images were collected at 5-15 s intervals using Laser TIRF 3 (Carl Zeiss) with an iXon3 EMCCD camera (Andor Technology). Mg-ATP-actin (15%-50% Oregon-green-actin) was mixed with recombinant proteins and 2 × TIRF buffer (20 mM imidazole pH 7.4, 0.4 mM ATP, 2 mM EGTA, 2 mM $MgCl_2$, 10 mM DTT, 100

mM KCl, 40 mg/ml catalase, 30 mM glucose, 200 mg/ml glucose oxidase, and 1% methylcellulose 4000 cP ). The mixture was injected into a flow cell and images were acquired as soon as the focal plane was found at room temperature.

**Methods S6 MT and AF bundling activity**

Taxol-stabilized rhodamine-conjugated MTs (1 μM) or Alex-488 phalloidin-labeled AFs (1 μM) were incubated with 500 nM FH1FH2 at room temperature for 30 min, and then added in Alex-488 phalloidin-labeled AFs (1 μM) or taxol-stabilized rhodamine-conjugated MTs (1 μM), respectively. After incubating for 30 min or 4 h, 5 μL diluted samples were then placed on a slide and visualized using an LSM510 laser scanning confocal microscope.

## REFERENCES

1 Li, Y. *et al.* The type II Arabidopsis formin14 interacts with microtubules and microfilaments to regulate cell division. *The Plant cell* **22**, 2710-2726, doi:10.1105/tpc.110.075507 (2010).
